# Detecting tipping points of complex diseases by network information entropy

**DOI:** 10.1093/bib/bbae311

**Published:** 2024-07-03

**Authors:** Chengshang Lyu, Lingxi Chen, Xiaoping Liu

**Affiliations:** Key Laboratory of Systems Health Science of Zhejiang Province, School of Life Science, Hangzhou Institute for Advanced Study, University of Chinese Academy of Sciences, 1 Xiangshan Branch Alley, Xihu District, Hangzhou 310024, China; Department of Biomedical Sciences, City University of Hong Kong, 31 To Yuen Street, Kowloon Tong, Kowloon, Hong Kong 999077, China; Department of Biomedical Sciences, City University of Hong Kong, 31 To Yuen Street, Kowloon Tong, Kowloon, Hong Kong 999077, China; Key Laboratory of Systems Health Science of Zhejiang Province, School of Life Science, Hangzhou Institute for Advanced Study, University of Chinese Academy of Sciences, 1 Xiangshan Branch Alley, Xihu District, Hangzhou 310024, China

**Keywords:** dynamic network biomarkers (DNB), tipping point, sample-specific network (SSN), entropy, perturbed network

## Abstract

The progression of complex diseases often involves abrupt and non-linear changes characterized by sudden shifts that trigger critical transformations. Identifying these critical states or tipping points is crucial for understanding disease progression and developing effective interventions. To address this challenge, we have developed a model-free method named Network Information Entropy of Edges (NIEE). Leveraging dynamic network biomarkers, sample-specific networks, and information entropy theories, NIEE can detect critical states or tipping points in diverse data types, including bulk, single-sample expression data. By applying NIEE to real disease datasets, we successfully identified critical predisease stages and tipping points before disease onset. Our findings underscore NIEE’s potential to enhance comprehension of complex disease development.

## Introduction

For most biological processes, changes are not static or constant over time but tend to be dramatic, potentially triggered by minor intrinsic or extrinsic perturbations. These changes lead to irreversible and substantial alterations in systems.

The dynamic network biomarkers (DNB) method is a novel concept designed to capture the dynamic changes of the complex systems on the progress of disease transitions [1, 2]. Unlike traditional biomarkers that only measure static levels of molecular activity, DNB can reveal the critical state and the driving networks of diseases before clinical manifestations. DNB and related algorithms have been successfully applied to various diseases such as cancer, diabetes, and Alzheimer’s disease [3–8].

The application of DNB in understanding the progression of complex diseases has gained prominence in various studies. In the context of hepatocellular carcinoma, a dynamic cascaded method reconstructed dynamic gene networks from sample-based transcriptional data based on two biologically plausible assumptions that can characterize the dynamics and continuity of gene transcription [9]. A study identified two DNBs that indicate the occurrence of two severe exacerbations of disease and can be used to predict impending abrupt changes during the progression of type 1 diabetes [3]. In another study on type 2 diabetes, Sun et al. proposed a modification of DNB, differential expression network, to conduct a spatiotemporal analysis of type 2 diabetes [10], which reflects phenotypic differences at the network level. Liu *et al.* introduced the concept of information entropy into DNB. They defined a state-transition-based local network entropy using high-throughput data from a few samples to identify critical transitions during disease progression and early-warning signals of their dominant networks [11].

In the DNB theoretical framework, a complex disease’s progression is typically observed through three distinct stages [1]: (i) normal state, which is a stable and resilient condition—a person in this state is healthy; (ii) predisease state, which can be compared to a cliff, indicates a catastrophic turning point from normal to disease state with the potential to return to the normal state; (iii) disease state, which is another stable but undesirable condition—a person in this state is extremely sick and cannot return to a healthy state by self-alignment. In the pre-state or at the tipping point, a local module (group) of genes will exhibit increased fluctuations and correlations. And three variables can quantitatively describe these: (i) ${PCC}_{in}$, which means the Pearson correlation coefficient within a local group; (ii) ${PCC}_{out}$, which refers to the correlation between a local group and other genes; (iii) ${SD}_{in}$, which means the fluctuations (standard deviation) within a local group. Furthermore, at the time of qualitative change, the local ${PCC}_{in}$ and ${SD}_{in}$ will increase sharply. Thus a local or dominant group can be regarded as the DNB of the disease [1]. From another perspective, when analyzing time-related data, a local group with strong fluctuations and high correlation that emerges at a certain moment may signify the transition and the turning point from pre-disease state to disease state [3, 12]. The quantitative description of these three elements enables the early-warning signals to be more accurately reflected and distinguishes the key elements of DNB.

Sample-specific network (SSN) is a novel method based on single sample differential association information calculation [13]. Unlike differential expression analysis, SSN constructs a unique network for each sample with its molecular expression in different human diseases at the network level. Remarkably, SSN enhances the granularity of diagnosis and early-warning signals, detects the critical states of complex diseases in individual samples, as well as identifies individual-specific disease modules and driver genes [12, 13]. Additionally, SSN can discover new individual-specific driver genes and network patterns in various cancer types. It can also identify drug-resistance genes that may not exhibit significant differential expression between samples with or without drug resistance [13]. Overall, SSN accurately and effectively identifies critical states in individual samples, thus advancing the field of personalized medicine and precision medicine in the context of DNB.

Information entropy is a concept widely utilized in information theory, physics, statistics, and other disciplines to quantify the uncertainty or content of information. In the field of bioinformatics, information entropy also has various applications, including the analysis of early phenotypic changes in cancer using maximum thermodynamic entropy [14], the exploration of relationships between biomolecules using the thermodynamic entropy of the non-equilibrium system [15], the study of topological entropy of DNA sequences [16], the investigation of entropy and information in gene interaction networks [17], and so on.

**Figure 1:**
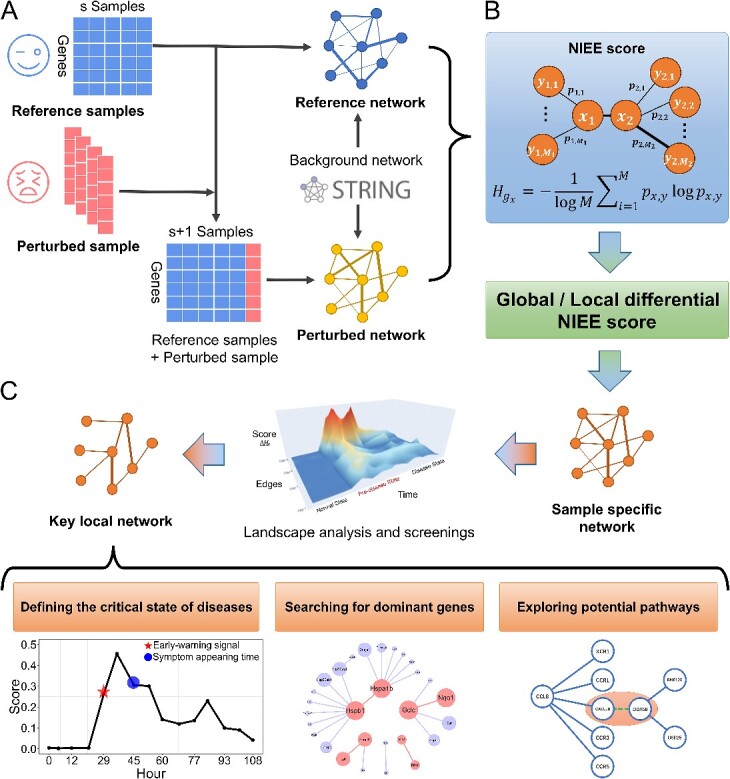
Network information entropy of edges (NIEE) workflow. (A) Upon obtaining multiple reference samples and a single perturbed sample from a designated dataset, we assemble matrices and networks for these sample data. Subsequently, by creating correlation networks (such as the Pearson correlation coefficient of gene expression), we filter through a background network (such as StringDB) within a certain threshold to obtain the reference and perturbed network. (B) The core algorithm of NIEE calculates the reference and perturbed network to derive the local differential NIEE score for one edge of a single sample and the global differential NIEE score for a general view of the sample-specific network. (C) Following a landscape analysis and threshold setting, we can identify the spontaneously formed network of filtered edges as the key local network and use it for further exploration.

In this paper, we proposed an enhanced method, Network Information Entropy of Edges (NIEE), to identify critical transitions and detect disease exacerbation signals from an edge-based perspective (Fig. 1). NIEE calculated the network information entropy of local edges by considering the interdependent regulation among genes. Subsequently, fluctuating samples are compared to reference samples or control groups from healthy individuals (Fig. 1A). This dynamic assessment enables us to gauge the sample’s status within the context of disease progression.

Specifically, NIEE, a DNB-based algorithm, offers notable advantages. (i) Unlike traditional biomarkers for disease determination, NIEE can start from a single-sample perspective and dynamically analyze an individual’s disease progression based on reference samples (Fig. 1A). It can detect pre-disease states and provide early-warning signals for diseases. (ii) NIEE utilizes entropy to measure the volatility of individual data, constructs the network using edges as fundamental components to broaden the information of genetic first-order and second-order neighbors, and improves the understanding of the entire network (Fig. 1B). (iii) NIEE is a model-free algorithm requiring only a suitable background network without any prior training on extensive data. (iv) Through the landscape analysis and a series of screenings (Fig. 1C), NIEE can identify some specific edges to construct self-forming networks, thereby facilitating the development of network markers and revealing the unknown gene relationships.

NIEE was also assessed on various real-world datasets, such as influenza, acute lung injuries, cancers, and pancreatic diseases. The results showed that NIEE could effectively predict upcoming diseases and show early-warning signals. We performed enrichment analysis on the key local network NIEE identified, revealing significant associations between biological functions and diseases in the experimental samples.

NIEE is an effective method for complex disease prediction and analysis based on three concepts: DNB, SSN, and information entropy. It offers a brand-new way to identify the pre-disease state, provide early-warning signals, and construct the key local network leading to potential disease. By evaluating and analyzing every single sample, NIEE exhibits substantial potential for clinical applications, especially in the realm of personalized disease prediction.

## Materials and methods

For utilizing NIEE, a sample set is required as a reference, which can be obtained from healthy individuals, normal tissue, or precancerous tissue. The next steps involve calculating the NIEE score for each sample through the following procedure.

### Constructing the background network

The background network ${N}_{BG}$ (Fig. 1A) is downloaded from the StringDB database (https://www.string-db.org) with a confidence score $\ge 0.85$ and any isolated node disconnects from the entire network is excluded from consideration.

### The local NIEE score

Once the background network ${N}_{BG}$ is obtained, our attention shifts to every edge in the entire network. Each edge serves as a bridge connecting two nodes (genes or proteins), and every single edge can be regarded as the smallest network (Fig. 1A).

The first-order neighbors of the two nodes in an edge could be denoted as ${E}_{x,y}\ \left(x=1,2;y=1,2,\dots, M\right)$, where $x$ corresponds to the two nodes (genes) forming the edge, and $y$ means the first-order neighbors of one node in the edge, totaling $M$.

Based on total $s$ samples, ${H}_E(s)$ in Eqn. (1) is used to calculate the local NIEE score for an edge and ${SD}_E(s)$ in Eqn. (2) is used to calculate the local standard deviation for the same edge.


(1) \begin{equation*} {H}_E(s)=\frac{\alpha }{\alpha +\beta}\cdotp{H}_{g_1}(s)+\frac{\beta }{\alpha +\beta}\cdotp{H}_{g_2}(s) \end{equation*}

(2) \begin{equation*} S{D}_E(s)=\frac{\alpha }{\alpha +\beta}\cdotp S{D}_{g_1}(s)+\frac{\beta }{\alpha +\beta}\cdotp S{D}_{g_2}(s) \end{equation*}

In Eqn. (1), the weights of nodes whose values $\alpha$ and $\beta$ are considered as the two parts of the edge. Here, we consider the degree of a node as its weight (Fig. 1B). While ${H}_{g_x}(s)$ and ${SD}_{g_x}(s)$ represent the information entropy and standard deviation for the specific node $x$ with total $s$ samples in the edge respectively, and these two parameters will be detailed in subsequent formulas. The Eqn. (3) represents the network entropy of one node in ${E}_{\left(x,y\right)}$, with $x=1,2$ corresponding to the two respective nodes.


(3) \begin{equation*} {H}_{g_x}(s)=-\frac{1}{\log M}\sum \limits_{y=1}^M{p}_{x,y}(s)\log{p}_{x,y}(s) \end{equation*}

Here, ${p}_{x,y}$ represents the correlation coefficient between node (gene) $x$ and $y$, while $M$ denotes the total number of first-order neighbors for the specific node $x$. The more detailed calculation steps for Eqn. (1)–(3) are further elucidated in the following Eqn. (4)–(6).


(4) \begin{equation*} {p}_{x,y}(s)=\frac{\left| PC{C}_{x,y}(s)\right|}{\sum_{y=1}^M\left| PC{C}_{x,y}(s)\right|} \end{equation*}

(5) \begin{equation*} PC{C}_{x,y}(s)=\frac{\sum_{n=1}^s\left({X}_n-\overline{X}\right)\left({Y}_n-\overline{Y}\right)}{\sqrt{\sum_{n=1}^s{\left({X}_n-\overline{X}\right)}^2}\sqrt{\sum_{n=1}^s{\left({Y}_n-\overline{Y}\right)}^2}} \end{equation*}

(6) \begin{equation*} S{D}_{g_x}(s)=\sqrt{\frac{\sum_{n=1}^s{\left({X}_n-\overline{X}\right)}^2}{s-1}} \end{equation*}

In Eqn. (4)–(6), ${X}_n$ means the expression level of gene $x$ in the ${n}^{th}$ sample, and $\overline{X}$ means the average expression level of gene $x$ in $s$ samples (same as $Y$). The Pearson Correlation Coefficient (*PCC*) is calculated by gene expression between one of the edge’s member genes and its first-order adjacency gene $y$ in total $s$ samples. The ${PCC}_{x,y}(s)$ is used to calculate the probability weights ${p}_{x,y}(s)$ by the network entropy ${H}_{g_x}(s)$. ${H}_{g_x}(s)$ means network entropy of gene ${g}_x$ in $s$ samples. And in Eqn. (6), ${SD}_{g_x}(s)$ means standard deviation of gene ${g}_x$ in $s$ samples.

### Constructing the perturbed network

The local NIEE score ${H}_E(s)$ and the local standard deviation ${SD}_E(s)$ are calculate based on the reference samples. Following the calculation of the local NIEE score and local standard deviation on reference samples, we add a single perturbed sample to the reference samples. This allows us to calculate the perturbed local NIEE score ${H}_E\left(s+1\right)$ in Eqn. (7) and perturbed local standard deviation ${SD}_E\left(s+1\right)$ in Eqn. (8).


(7) \begin{equation*} {\displaystyle \begin{array}{l}{H}_E\left(s+1\right)=\frac{\alpha }{\alpha +\beta}\cdotp{H}_{g_1}\left(s+1\right)+\frac{\beta }{\alpha +\beta}\cdotp{H}_{g_2}\left(s+1\right)\\{}=-\frac{\alpha }{\alpha +\beta}\cdotp \frac{1}{\log M}\sum \limits_{y=1}^M\left[{p}_{1,y}\left(s+1\right)\log{p}_{1,y}\left(s+1\right)\right]\\{}-\frac{\beta }{\alpha +\beta}\cdotp \frac{1}{\log M}\sum \limits_{y=1}^M\left[{p}_{2,y}\left(s+1\right)\log{p}_{2,y}\left(s+1\right)\right]\end{array}} \end{equation*}

(8) \begin{equation*} S{D}_E\left(s+1\right)=\frac{\alpha }{\alpha +\beta }S{D}_{g_1}\left(s+1\right)+\frac{\beta }{\alpha +\beta }S{D}_{g_2}\left(s+1\right) \end{equation*}

(9) \begin{equation*} \left\{ sampl{e}_1, sampl{e}_2,\dots, sampl{e}_s, sampl{e}_{perturbed}\right\}, \end{equation*}

where $s+1$ refers to $s$ reference samples concatenated with one perturbed sample, as illustrated in Eqn. (9) (Fig. 1A, B). The calculation of ${p}_{x,y}\left(s+1\right)$ and ${SD}_{g_x}\left(s+1\right)$ follows the same procedure as detailed in Eqn. (3)–(6).

### The differential local NIEE score

After getting all the perturbed local NIEE scores with every perturbed sample, we can define the differential local NIEE score $\triangle{H}_E\left(s+1\right)$ in Eqn. (10) for every edge $E$ (Fig. 1B).


(10) \begin{equation*} \triangle{H}_E\left(s+1\right)=\left|{SD}_E\left(s+1\right)-{SD}_E(s)\right|\cdotp \left|{H}_E\left(s+1\right)-{H}_E(s)\right| \end{equation*}

In Eqn. (10), $\triangle{H}_E\left(s+1\right)$ represents the product of the absolute value of $SD$ and the absolute value of the local edge entropy. It denotes the fluctuation of the edge in the perturbed sample compared to the reference samples.

### The key differential local NIEE score and global differential NIEE score

In the case of a perturbed sample, we derive a value termed the key differential local NIEE score, and this score encapsulates the primary situation of a perturbed sample observed via a key local network (i.e. key sub-network) filtered based on a specific threshold score for all edges. On a broader scale, a comprehensive assessment can be carried out using all edges to calculate the global differential NIEE score to get a general state of a perturbed sample.

Here, the key differential local NIEE score $\triangle LH\left(s+1\right)$ and the global differential NIEE score $\triangle GH\left(s+1\right)$ are presented in Eqn. (11)–(12),


(11) \begin{equation*} \triangle LH\left(s+1\right)=\frac{1}{WL}\sum \limits_{e=1}^{WL}\left[\triangle{H}_e\left(s+1\right)\right] \end{equation*}

(12) \begin{equation*} \triangle GH\left(s+1\right)=\frac{1}{WG}\sum \limits_{e=1}^{WG}\left[\triangle{H}_e\left(s+1\right)\right] \end{equation*}

where $WL$ means all edges in the key local network, and $WG$ means all edges in the whole network.

## Results

The comprehensive utilization of various omics data in conjunction with NIEE across diverse experimental samples allowed us to pinpoint critical tipping points and early-warning signals in a spectrum of complex diseases. These revelations include the identification of key edges and a distinct key local network, particularly during moments marked by a rapid increase in NIEE scores. This emergent key local network can be conceptualized as a novel DNB, holding potential significance in diagnosing individuals on the brink of illness.

### Data extraction

In our investigation of time-series datasets, we thoroughly examined short-term datasets such as GSE30550 [18], GSE52428 [19], and GSE73072 [20] to study flu-related early-warning signals and GSE2565 [21] for acute lung injury. To better understand chronic diseases, we delved into long-term time-series datasets, specifically LUAD, from the TCGA database [22]. Moreover, our innovative exploration extended to pancreatic lymph node datasets of NOD mice (GSE15150) [23], emphasizing the robustness of our method. All GSE-numbered datasets can be accessed on the NCBI GEO database (https://www.ncbi.nlm.nih.gov/geo/), while the TCGA database is available through the National Cancer Institute (https://portal.gdc.cancer.gov/).

To construct the background network ${N}_{BG}$, we utilized the *Homo sapiens* and *Mus musculus* networks from the StringDB database (https://www.string-db.org/, version 11.5).

In the following section, we provide detailed insights into the specific workflows and results of the NIEE algorithm applied to these datasets. The noteworthy early-warning results serve as compelling evidence for the NIEE algorithm’s efficacy in assessing individual health status and identifying critical tipping points in disease progression.

### Detecting the early-warning signals and the implicit information in the key local network of influenza A H3N2

**Figure 2:**
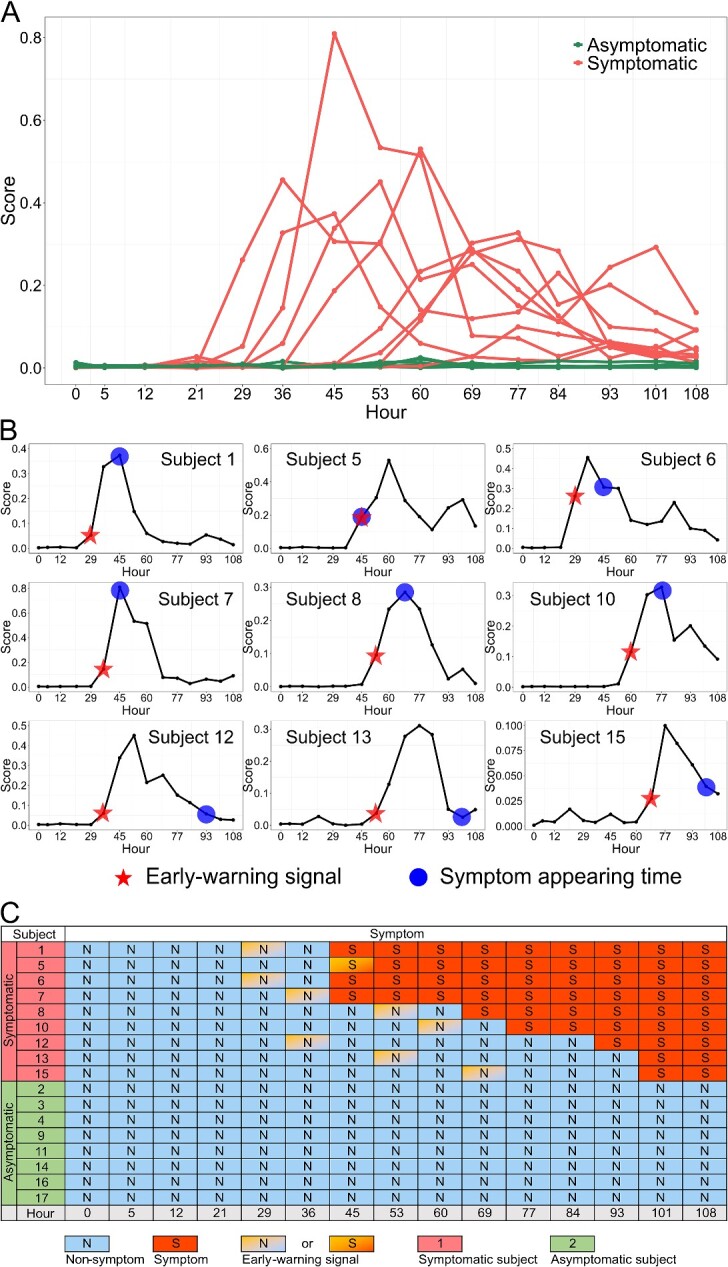
Identification of the pre-disease states of symptomatic subjects infected with H3N2 (GSE30550) using the NIEE algorithm. (A) Line graph of NIEE scores for all subjects, with the groups of symptomatic subjects and asymptomatic subjects. (B) Specific NIEE score line graphs for the nine symptomatic subjects. The pentagrams signify the moment when the subject’s early-warning signal appeared, indicating the NIEE-determined critical pre-disease state. The circles represent the moment when the subjects showed clinical symptoms of influenza. (C) Biological temporal table of NIEE personalized early-warning signals and clinical diagnoses for all subjects.

In dataset GSE30550, 17 healthy humans were injected with the living H3N2 virus, and their gene expression data were collected from blood at sixteen time points over 132 hours (−24, 0, 5, 12, 29, 36, 45, 53, 60, 69, 77, 84, 93, 101, 108 hours) (Fig. 2C). After injecting the virus at 0 hours, 9 of 17 subjects developed flu symptoms (subjects 1, 5, 6, 7, 8, 10, 12, 13, 15), and the rest remained asymptomatic (subjects 2, 3, 4, 9, 11, 14, 16, 17). The samples from all subjects at the −24-hour time point were considered as the reference samples group, representing a healthy state unaffected by the virus.

Following the workflow of the NIEE algorithm, a background network was constructed based on StringDB with a cutoff of 0.85. Differential local NIEE scores were calculated for each subject at 15 time points (Fig. 2A). The top 1% edges in the differential local NIEE scores of all symptomatic objects at each time point were screened out, and a key local network, comprising 13 key edges, was identified based on the frequency of their occurrence (Table S1). We regarded the sum of the key local network’s score as the key differential local NIEE score ($\triangle LH\left(s+1\right)$) to make further quantitative analysis.

There is a significant difference in key differential local NIEE score (Fig. 2A) between clinically symptomatic subjects (red line chart) and asymptomatic subjects (green line chart). Evidently, a sharply increasing NIEE score denotes the tipping point where significant changes are about to occur, and for the symptomatic subjects, it means that clinical symptoms are around the corner. All the symptomatic subjects had early-warning signals, and eight of them had NIEE early-warning signals before clinical symptoms appeared (Fig. 2B and 2C). In contrast, none of the asymptomatic subjects showed early-warning signals (Fig. 2A–C). For the H3N2 influenza virus, the key local network screened by the NIEE algorithm effectively identifies samples before the appearance of clinical symptoms and provides precise individualized early-warning signals for everyone (Fig. 2B). Furthermore, we can use the key local network on other samples or patients for disease prediction.

**Figure 3:**
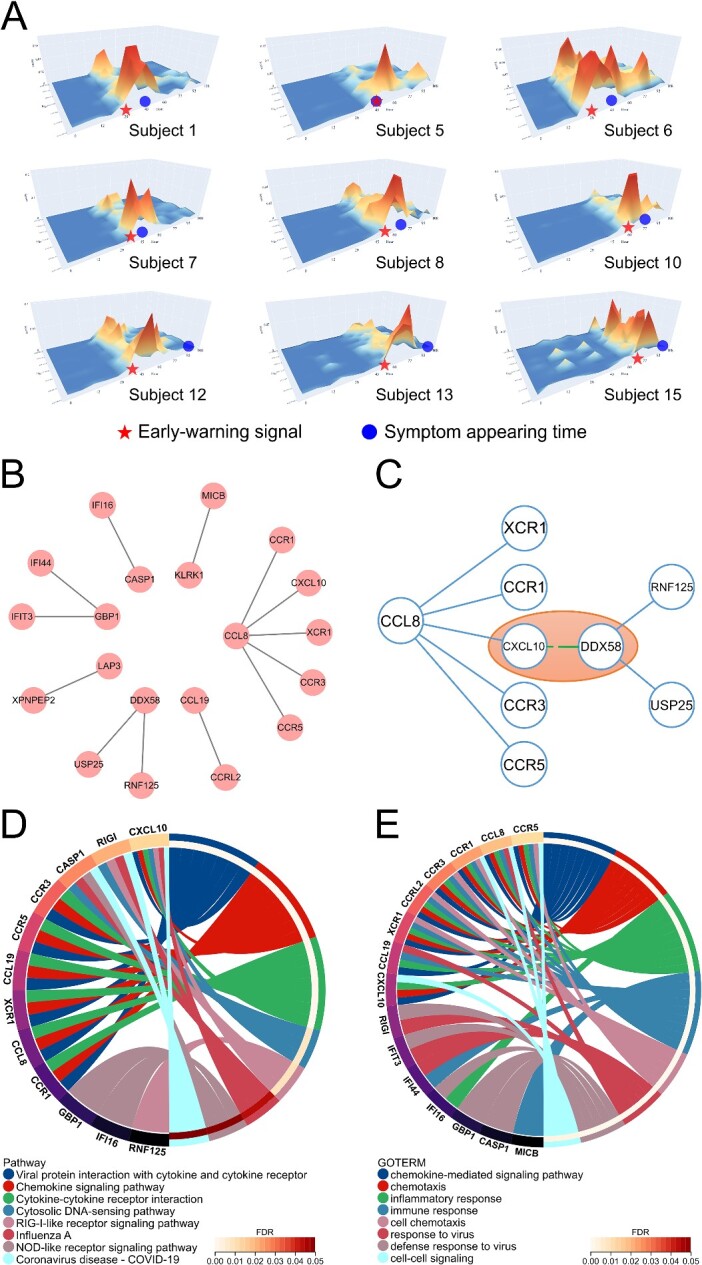
In-depth analysis of 13 edges in the key local network of Influenza A H3N2 (GSE30550). (A) Landscape graphs of NIEE scores for the key local networks in nine symptomatic subjects. The X-axis represents time (hour), the Y-axis represents edge (gene–gene), and the Z-axis represents the NIEE score of each edge. The pentagrams indicate the moment of the subject’s early-warning signal, implying the NIEE-determined critical pre-disease state. The circles represent the moment when the subjects showed clinical symptoms of influenza. In the landscape graph of each symptomatic subject, the peaks usually appear in patches, implying that there is no singular edge in the key local network that attains an extremely high score, but rather the joint effect of multiple edges to affect the overall score. (B) Network structure graph illustrating the self-forming network composed of all edges in the key local network. (C) Exploration of potential members in the pathway of Influenza A and identification of negative regulation between CXCL10 and DDX58. (D and E) Enrichment analysis circle graphs presenting all genes in the key local network of GSE30550. The left side of the circle displays each gene, while the right side depicts the associated biological process or pathway. (D) Biological process enrichment of all genes in the key local network in GO process. (E) Pathway enrichment of all genes in the key local network in the KEGG pathway.

The landscape graph (Fig. 3A) and key local network graph (Fig. 3B) illustrate the network characteristics of the close connections between the various genes in the key local network identified by the NIEE algorithm. The landscape graph (Fig. 3A) corresponding to each symptomatic subject displayed the scores of each edge in the key local network in a 3D form. It not only revealed that the key local network consists of multiple edges, without any single edge having an extremely high score that significantly influences the overall differential local NIEE score, but also indicated that the NIEE algorithm uses a broader perspective to evaluate the current state by the overall network.

In the early-warning signals provided by NIEE for each subject, there were no false-positive warning signals compared with the previous method (no signal versus three signals) [24], and only one symptomatic subject’s early-warning signal coincided with the disease onset moment (one subject versus two subjects) [25].

To demonstrate the effectiveness of NIEE in detecting disease early-warning signals and gene function associations, enrichment analysis tools in Database for Annotation, Visualization, and Integrated Discovery (DAVID, https://david.ncifcrf.gov/) were utilized to analyze the genes in the key local network. These genes were enriched in several biological processes or functions, such as immunity, virus, chemokine, cytokine, and influenza (Fig. 3D, E, and Table S2). This suggests that the key local network not only identified the pre-disease state before the onset of influenza symptoms but was also effective in disease early warning, indicating its involvement in one or more parts of the biological processes related to influenza virus infection.

While only three of the genes were annotated in the KEGG Influenza A pathway [26], the remaining genes were only related to more general keywords such as cytokines and immunity (Fig. 3C). We tried to use the key local network to explore more potential gene relationships.

CCL8 was a chemokine with the highest degree in the key local network of genes we identified, which can bind to various chemokine receptors and recruit monocytes, lymphocytes, basophils, and eosinophils. CCL8 may also involve neoplasia and inflammatory host responses [27]. In our key local network, CCL8 had five links to other genes: CXCL10, XCR1, CCR1, CCR3, and CCR5. These links can be classified into two pathways: cytokine-cytokine receptor interaction and viral protein interaction with cytokine and cytokine receptor [28].

Further literature review revealed that CCL8 interacts with receptors CCR1, CCR5 [29, 30], and CCR3 [31], expressed on various cell types, including granulocytes, mononuclear phagocytes, and T lymphocytes. High expression levels of CCR1 and CXCL10 were detected in various cells, such as alveolar macrophage and NK cells, in the mice at the initial stage of the influenza A virus infection experiment [32]. Similarly, high expression levels of CCL8 and CXCL10 were noted in ferret lungs during the first and the second days of H1N1 2009 influenza A virus infection [33].

Based on the high score between CCL8 and CCR1, CCR3, and CCR5 by NIEE, we believe that the interaction of CCL8 with these C-C motif chemokine receptors may represent a critical pathway in the early stage of influenza A infection. Moreover, CCL8 may have a potential interaction with CXCL10, which has been identified as an important gene of early infection in the influenza A pathway [34]. Meanwhile, in the simulation result of ClusPro (protein–protein docking software, https://cluspro.bu.edu/), CXCL10 (PDB: 1O80) - CCL8 (PDB: 1ESR) had 84 and 72 members in the two clusters with the highest number of members. In these two clusters, the weighted scores for the center and lowest energy are −828.7, −837.0, and − 937.2, −937.2, respectively [35, 36].

In the sub-network consisting of DDX58 (RIG-I), RNF125, and USP25 (Fig. 3C), enrichment analysis indicated their association with the RIG-I-like receptor signaling pathway [28]. This trio of genes collaboratively formed a sub-network intricately involved in orchestrating the innate immune response to viral infections. DDX58, functioning as an innate immune receptor, plays a pivotal role in detecting cytoplasmic viral nucleic acids. Its activation sets off downstream signaling cascades, ultimately producing type I interferons and pro-inflammatory cytokines. Among the final synthetic products in this pathway is CXCL10, a chemokine ligand [28], and DDX58 (PDB: 8DVU) - CXCL10 (PDB: 1O80) had potential interaction in ClusPro’s simulation with 81 members as well as −1405.2 weighted score of lowest energy [35, 36]. USP25, identified as a deubiquitinating enzyme, is crucial in maintaining a delicate balance. It cleaves ubiquitin chains from key players such as RIG-I, TRAF2, and TRAF6, inhibiting SEV-induced type I IFN signaling [37]. Conversely, RNF125 serves as a ubiquitin ligase that negatively regulates the RIG-I signaling pathway. Its role involved promoting the degradation of RIG-I, as demonstrated in previous studies [38]. The coordinated actions of these genes within the sub-network emphasized the nuanced regulatory mechanisms governing the RIG-I-like receptor signaling pathway during the innate immune response to viral infections.

NIEE detected two edges (DDX58-RNF125 and DDX58-USP25) with significant fluctuations in this sub-network, suggesting crucial connections in the initial infection stage of a hypothetical influenza pathway that negatively regulates RIG-I receptors and cytokines.

To further verify the excellent effect of NIEE on single-sample time-series datasets, we applied it to three additional datasets [19, 21, 39] for early-warning signal and tipping point detection in H3N2 Influenza A, HRV, and acute lung injury in Notes S1–S3 and Tables S3–S6.

In essence, NIEE, by quantifying network fluctuations through the lens of edges, provides not only early-warning signals for impending symptoms and disease onset but also reveals valuable insights into key networks and their edges. This has implications for understanding gene interactions, biological processes, pathways, pathogenesis, and drug research.

### Identifying early-warning signals and key gene networks for the deterioration and metastasis of LUAD

Different from previous acute diseases, cancer is typically a chronic disease with a long-time span, and metastasis is a primary cause of death in malignant cancers. Especially the 5-year survival rate for patients with distant metastases remains extremely low [40].

Normally, cancer spreads to distant parts of the body, including the involvement of the liver or bone marrow, lungs, or cerebrospinal fluid at Stage IV [41]. However, for many unfortunate patients, cancer is often diagnosed only after it has metastasized or when various obvious discomforts have already occurred. NIEE aims to detect the early-warning signals before cancer deterioration and metastasis.

In this section, we applied the NIEE algorithms to the TCGA-LUAD dataset, which comprises 518 tumor samples ranging from Stage IA to Stage IV (Table S8). We compared these tumor samples with 14 Stage IA tumor-adjacent samples, serving as the reference group (or control group), to discover the early-warning signal for metastasis and the key local network influencing tumor deterioration.

Unlike time series data, each sample has no temporal continuity in LUAD datasets. Thus, we calculated the differential local NIEE score and global differential NIEE score for every sample, then averaged the values for samples in the same stage as the quantitative score to measure cancer metastasis and progression.

**Figure 4:**
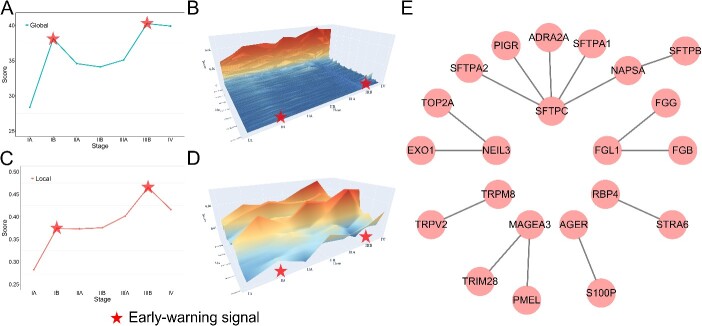
In-depth analysis of the global network and 15 edges in the key local network of LUAD. (A) Line graphs of NIEE scores for the global network. (B) Landscape graph illustrating NIEE scores for the global network. (C) Line graphs of NIEE scores for the key local network. (D) Landscape graph depicting NIEE scores for the key local network. (E) Network structure graph showcasing the self-forming network constituted of all edges in the key local network.

From the global differential NIEE score, we identified two tipping points for LUAD in Stages IB and IIIB (Fig. 4A and B). These critical signals might denote important transitions in cancer deterioration.

Furthermore, we screened the differential local NIEE score in each stage and selected the top 15 edges (Table S9) based on the frequency of occurrence as the key local network. Stage III is the highest point among all stages (Fig. 4C), with two sharp increases in Stages IB and IIIB and a significant decline in Stage IV (Fig. 4C). To obtain a comprehensive view and discover dominant edges in the key local network, we draw a landscape plot for every edge in the key local network (Fig. 4D). Most edges changed rapidly and showed early-warning signals at Stages IB and IIIB. In the self-forming graph (Fig. 4E), the edges we found are not all independent but can spontaneously form multiple small sub-networks. These small sub-networks may imply potential close connections between genes and joint effects on cancer progression.

Next, we employed two distinct grouping methods for survival analysis based on disease duration and score.

**Figure 5:**
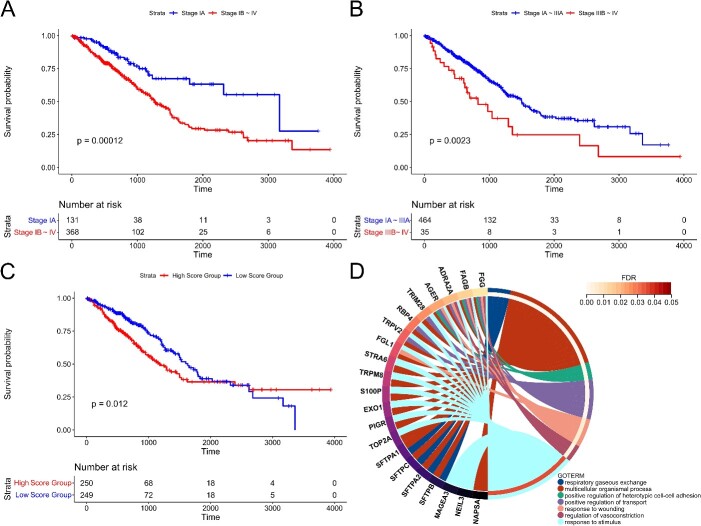
Survival and enrichment analysis of LUAD (A) survival analysis based on the stage status, with individuals categorized into Stage IA and Stages IB–IV groups. (B) Survival analysis grouping individuals into Stages IA–IIIA and Stage IIIB–IV. (C) Survival analysis based on NIEE scores, classifying individuals into the top 50% and bottom 50% groups. (D) Biological process enrichment of the key local network for LUAD in GOTERM biological process.

Considering the stage of cancer progression and the tipping point found by NIEE, we divided the samples into four groups by the early-warning signals: Stage IA, Stages IB ~ IV, Stages IA ~ IIIA, and Stages IIIB ~ IV. The first two groups exhibited a highly significant *p*-value of 0.00012 (Fig. 5A), while the last two groups had a p-value of 0.0023 (Fig. 5B). This indicated that the prognosis of patients before the early-warning signals was markedly better than the latter, and NIEE has a certain effect on the early warning of cancer progression and metastasis.

Then, we divided samples into two groups based on their scores, the top 50% and the bottom 50%, and analyzed the total score and partial edges scores of these groups. In comparing these two groups, the survival probability of the high-scoring group was significantly lower than that of the low-scoring group at the same survival time, with a significant *P*-value of .012 (Fig. 5C). This illustrates that the NIEE score can not only quantitatively measure the early-warning signal but also reflect the relationship between the NIEE score, disease severity, and survival duration.

Subsequently, we conducted enrichment and survival analyses on the 15 key edges identified as crucial network biomarkers. Our study revealed that the genes in the key local network are partially enriched in various keywords such as lung, breathing, and trachea (Fig. 5D and Table S10). This aligns closely with our organ-level study of lung adenocarcinoma. Notably, the enrichment analysis of Jensen Lab Disease-gene associations [42] highlighted associations with lung diseases. Additionally, the keyword ‘Positive regulation of heterotypic cell-cell adhesion’ strongly correlates with cancer metastasis. Literature reports indicate that disruptions in cell adhesion can guide tumor cells to specific tissues, facilitating arrest, migration across vessel walls, and growth at secondary sites [43, 44]. A more detailed discussion of the genes in the key local network can be found in Note S4.

Collectively, our multiple analyses underscore the close association of most genes in the key local network with the deterioration and metastasis of LUAD, evident in both gene function and survival analysis. The first early-warning signal may be a critical time point of cancer progression from early to advanced stages, and the second early-warning signal appears to be a potent indicator of imminent cancer metastasis and spread. We are confident that implementing NIEE on LUAD data not only effectively signals warnings during the ongoing cancer deterioration but also has promising potential for developing warning systems in various other types of cancer in the future.

### Further exploration—application of NIEE in destructive insulitis and hyperglycemia of NOD mice with incomplete information

NIEE was also applied to analyze the disease induction and progression dataset in NOD mice (GSE15150), which has some information loss in the contributor’s original processing. Due to space limitations, we discussed the detailed data processing and analysis process in Note S5.

## Discussion

One common approach in biological network analysis is constructing a fully connected network from each node and then reducing the network size by iteratively deleting nodes based on their degree. This method aims to assign a weighted score to each node, identifying the most crucial nodes in the network. However, this approach may need to pay more attention to the essential role of edges in the network and ignore the different possible connections between nodes when deleting a node and its associated edges.

In this study, we regard genes as nodes and their interactions as edges to build a large network. It can capture more information and has more precise connection information between each node, which helps us better identify the key local network. Especially in the background network, only a few end nodes have a single first-order neighbor, resembling leaves on a tree. As these nodes have a minimal impact on edges and the entire network, we eliminated these related edges. Based on these characteristics, NIEE provides a novel perspective and solution for early disease warning, aiming to reduce or prevent the subsequent risks and damages from disease progression or deterioration. In contrast to traditional biomarkers that distinguish whether a sample is diseased, NIEE integrates single-sample or multi-sample high-dimensional data to detect biological network fluctuations by differences in edges at various time points and displays early-warning signals before complex disease onset where there is no significant change in the organism. The source code for NIEE is now accessible on GitHub (https://github.com/lllvcs/NIEE).

We have also explored datasets that underwent preliminary data processing and may contain missing information. In the case of constructing a normal distribution matrix as a reference network in the data of destructive insulitis and hyperglycemia of NOD mice, we identified potential early-warning signals before the onset of the disease and screened key genes potentially affecting the disease based on the key local network. These results further indicate that the NIEE algorithm showcases high universality in data applications and exhibits a notable early-warning effect, even in the presence of missing information. Furthermore, delving into the genes within the key local network illuminates valuable insights into potential factors contributing to the induction of diabetes.

Besides, in Note S2, Fig. S3, and Tables S5–S6, NIEE was utilized to detect early-warning signals of Human Rhinovirus infection. In this additional dataset we examined, NIEE consistently demonstrated reliable early-warning signals, further substantiating the algorithm’s universality.

Moreover, when applying the NIEE algorithm to the analysis of RNA-seq data across various disease conditions and continuous time-series data from an individual sample, it serves as a powerful tool for health assessment, enabling personalized precision medicine. By monitoring dynamic changes in the score, we can identify the critical state or tipping point of biological processes or diseases and provide timely intervention. This versatile algorithm has potential applications to diverse types of biological data, including ChIP-seq data for chromatin-protein interactions, microbial population abundance data for microbial ecology, and metabolomics data for metabolic pathways. Additionally, due to NIEE’s novel model-free nature, extensive learning from a large number of datasets in the preliminary stages is not necessary, as is the case with machine learning. Instead, it requires the determination of specific thresholds. In our dataset practices, we selected a threshold of 0.85 for the StringDB background network, ensuring a high correlation between genes. Simultaneously, while filtering key local networks, we typically adopt a less than 1% threshold to select edges with higher scores. However, it is essential to acknowledge that such fixed threshold settings and background network selections may overlook potential connections between genes.

We have further explored and analyzed integrated background networks, correlation coefficients, and gene weights with the same GSE30550 dataset in Notes S6–S8 and obtained certain results. Our research also highlighted a comparative study between BioTIP [45], a pioneering disease early warning algorithm, and NIEE within the context of the lung adenocarcinoma dataset, as outlined in Note S9.

However, we believe that there is still much to be done. As Zitnik *et al.* have discussed in their outlook on future network biology [46], exploring more advanced and comprehensive methods for background network construction, screening, and developing better algorithms will be a focus of our future research. Inspired by their prospect, we see the integration of multimodal data with heterogeneous networks as a promising new field that warrants further exploration. In our future work, we plan to explore more diverse types of biological data, further screen and integrate more different types of biological networks and combine them with leading technologies such as graph neural networks to advance the field of disease early warning.

Furthermore, we intend to enhance and optimize the NIEE algorithm in future endeavors. We anticipate that it will be applied to a broader range of datasets, including single-cell [47, 48], cell differentiation [49], and cell fate choices [45]. Simultaneously, multi-omics data from the same type of disease can be integrated for further multi-dimensional analysis. This approach aligns with our goal of pushing the boundaries of what is possible in the realm of biological data analysis.

In conclusion, the NIEE algorithm, with its capacity to estimate the fluctuation states of individuals and groups, offers a novel perspective on understanding disease onset and progression. Our exploration involves in identifying a key local network and relevant biological dynamic network markers, shedding light on critical states and tipping points during disease development. Notably, various key genes within this network, each carrying distinct implications for disease pathogenesis, present promising avenues for further research. The intricate connections between these genes and their involvement in crucial biological processes underscore their significance. The NIEE’s contribution of valuable information not only aids in targeted therapeutic approaches but also holds immense potential for personalized disease prediction, early warning, diagnosis, and analysis. As validating the NIEE with various datasets, we anticipate its potential to enable early disease warnings across diverse types of illnesses. Additionally, discovering isolated edges within the key local network, though seemingly unrelated to current diseases, may serve as valuable insights for future exploration, potentially emerging as new biomolecular or network markers.

**Key Points**

- Network Information Entropy of Edges (NIEE) is a novel approach for detecting critical transitions or tipping points in the disease process and shows enormous potential for early diagnosis and intervention in complex diseases.
- NIEE applies network theory and information entropy to assess dynamic changes within sample-specific molecular interaction networks, offering an edge-focused perspective rather than traditional node-based analysis.
- NIEE identifies dynamic network biomarkers that serve as early-warning signals, particularly demonstrated through case studies in acute and chronic diseases.

## Supplementary Material

Supplementary_materials_final_bbae311

Supplementary_table_bbae311

## Data Availability

The datasets supporting the conclusions of this article can be downloaded at http://cancergenome.nih.gov/ and http://www.ncbi.nlm.nih.gov/geo/ with accession number: GSE30550, GSE52428, GSE17156, GSE2565, and GSE15150. To ensure reproducible results, the source codes are available at https://github.com/lllvcs/NIEE.

## Author Biographies

**Chengshang Lyu**, MEng, currently serves as a research assistant and a PhD student in the Department of Biomedical Sciences, City University of Hong Kong. He received his bachelor’s and master’s degrees from Xi’an Jiaotong University and the University of Chinese Academy of Sciences, respectively. He is interested in bioinformatics, deep learning, algorithm development, and design.

**Lingxi Chen**, PhD, currently serves as an assistant professor in the Department of Biomedical Sciences, City University of Hong Kong. She is interested in bioinformatics, deep learning, algorithm development, and design.

**Xiaoping Liu**, PhD, currently serves as a professor in the Key Laboratory of Systems Health Science of Zhejiang Province, School of Life Science, Hangzhou Institute for Advanced Study, University of Chinese Academy of Sciences, Hangzhou, China. He studies the fields of computational systems biology and bioinformatics.

## References

[1] Chen L, Liu R, Liu ZP. et al. Detecting early-warning signals for sudden deterioration of complex diseases by dynamical network biomarkers. Sci Rep 2012;2:342. 10.1038/srep00342. 22461973 PMC3314989

[2] Aihara K, Liu R, Koizumi K. et al. Dynamical network biomarkers: theory and applications. Gene 2022;808:145997. 10.1016/j.gene.2021.145997. 34626720

[3] Liu X, Liu R, Zhao XM. et al. Detecting early-warning signals of type 1 diabetes and its leading biomolecular networks by dynamical network biomarkers. BMC Med Genomics 2013;6:S8. 10.1186/1755-8794-6-S2-S8. PMC3654886 23819540

[4] Zhang C, Liu J, Shi Q. et al. Comparative network stratification analysis for identifying functional interpretable network biomarkers. BMC Bioinformatics 2017;18:48. 10.1186/s12859-017-1462-x. 28361683 PMC5374559

[5] Yang B, Li M, Tang W. et al. Dynamic network biomarker indicates pulmonary metastasis at the tipping point of hepatocellular carcinoma. Nat Commun 2018;9:678. 10.1038/s41467-018-03024-2. 29445139 PMC5813207

[6] Guo WF, Zhang SW, Zeng T. et al. Network control principles for identifying personalized driver genes in cancer. Brief Bioinform 2020;21:1641–62. 10.1093/bib/bbz089. 31711128

[7] Liu ZP, Wang Y, Zhang XS. et al. Identifying dysfunctional crosstalk of pathways in various regions of Alzheimer's disease brains. BMC Syst Biol 2010;4:S11. 10.1186/1752-0509-4-S2-S11. 20840725 PMC2982685

[8] Jiang L, Sui D, Qiao K. et al. Impaired functional criticality of human brain during Alzheimer's disease progression. Sci Rep 2018;8:1324. 10.1038/s41598-018-19674-7. 29358749 PMC5778032

[9] Zhu H, Rao RSP, Chen L. Reconstructing dynamic gene regulatory network for the development process of hepatocellular carcinoma. 2012 IEEE International Conference on Bioinformatics and Biomedicine Workshops. 2012:159–165. 10.1109/BIBMW.2012.6470298.

[10] Sun SY, Liu ZP, Zeng T. et al. Spatio-temporal analysis of type 2 diabetes mellitus based on differential expression networks. Sci Rep 2013;3:2268. 10.1038/srep02268. 23881262 PMC3721080

[11] Liu R, Li M, Liu ZP. et al. Identifying critical transitions and their leading biomolecular networks in complex diseases. Sci Rep 2012;2:813. 10.1038/srep00813. 23230504 PMC3517980

[12] Liu R, Yu X, Liu X. et al. Identifying critical transitions of complex diseases based on a single sample. Bioinformatics 2014;30:1579–86. 10.1093/bioinformatics/btu084. 24519381

[13] Liu X, Wang Y, Ji H. et al. Personalized characterization of diseases using sample-specific networks. Nucleic Acids Res 2016;44:e164. 10.1093/nar/gkw772. 27596597 PMC5159538

[14] Remacle F, Kravchenko-Balasha N, Levitzki A. et al. Information-theoretic analysis of phenotype changes in early stages of carcinogenesis. Proc Natl Acad Sci U S A 2010;107:10324–9. 10.1073/pnas.1005283107. 20479229 PMC2890488

[15] Borzou A, Sadygov RG. A novel estimator of the interaction matrix in graphical Gaussian model of omics data using the entropy of non-equilibrium systems. Bioinformatics 2021;37:837–44. 10.1093/bioinformatics/btaa894. 33067612 PMC8098027

[16] Koslicki D . Topological entropy of DNA sequences. Bioinformatics 2011;27:1061–7. 10.1093/bioinformatics/btr077. 21317142

[17] Wallace ZS, Rosenthal SB, Fisch KM. et al. On entropy and information in gene interaction networks. Bioinformatics 2019;35:815–22. 10.1093/bioinformatics/bty691. 30102349 PMC7245360

[18] Huang Y, Zaas AK, Rao A. et al. Temporal dynamics of host molecular responses differentiate symptomatic and asymptomatic influenza a infection. PLoS Genet 2011;7:e1002234. 10.1371/journal.pgen.1002234. 21901105 PMC3161909

[19] Woods CW, McClain MT, Chen M. et al. A host transcriptional signature for presymptomatic detection of infection in humans exposed to influenza H1N1 or H3N2. PloS One 2013;8:e52198. 10.1371/journal.pone.0052198. 23326326 PMC3541408

[20] Liu TY, Burke T, Park LP. et al. An individualized predictor of health and disease using paired reference and target samples. BMC Bioinformatics 2016;17:47. 10.1186/s12859-016-0889-9. 26801061 PMC4722633

[21] Sciuto AM, Phillips CS, Orzolek LD. et al. Genomic analysis of murine pulmonary tissue following carbonyl chloride inhalation. Chem Res Toxicol 2005;18:1654–60. 10.1021/tx050126f. 16300373

[22] Grossman RL, Heath AP, Ferretti V. et al. Toward a shared vision for cancer genomic data. N Engl J Med 2016;375:1109–12. 10.1056/NEJMp1607591. 27653561 PMC6309165

[23] Kodama K, Butte AJ, Creusot RJ. et al. Tissue- and age-specific changes in gene expression during disease induction and progression in NOD mice. Clin Immunol 2008;129:195–201. 10.1016/j.clim.2008.07.028. 18801706 PMC2592195

[24] Liu X, Chang X, Liu R. et al. Quantifying critical states of complex diseases using single-sample dynamic network biomarkers. PLoS Comput Biol 2017;13:e1005633. 10.1371/journal.pcbi.1005633. 28678795 PMC5517040

[25] Liu R, Chen P, Chen L. Single-sample landscape entropy reveals the imminent phase transition during disease progression. Bioinformatics 2020;36:1522–32. 10.1093/bioinformatics/btz758. 31598632

[26] Ouzounis CA, Khatri P, Sirota M. et al. Ten years of pathway analysis: current approaches and outstanding challenges. PLoS Comput Biol 2012;8(2):e1002375. 10.1371/journal.pcbi.1002375. PMC3285573 22383865

[27] Maglott D, Ostell J, Pruitt KD. et al. Entrez gene: gene-centered information at NCBI. Nucleic Acids Res 2007;35:D26–31. 10.1093/nar/gkl993. 17148475 PMC1761442

[28] Kanehisa M, Furumichi M, Sato Y. et al. KEGG for taxonomy-based analysis of pathways and genomes. Nucleic Acids Res 2023;51:D587–92. 10.1093/nar/gkac963. 36300620 PMC9825424

[29] Moser B, Loetscher P. Lymphocyte traffic control by chemokines. Nat Immunol 2001;2:123–8. 10.1038/84219. 11175804

[30] Ruffing N, Sullivan N, Sharmeen L. et al. CCR5 has an expanded ligand-binding repertoire and is the primary receptor used by MCP-2 on activated T cells. Cell Immunol 1998;189:160–8. 10.1006/cimm.1998.1379. 9790730

[31] Ge B, Li J, Wei Z. et al. Functional expression of CCL8 and its interaction with chemokine receptor CCR3. BMC Immunol 2017;18:54. 10.1186/s12865-017-0237-5. 29281969 PMC5745793

[32] Zhang J, Liu J, Yuan Y. et al. Two waves of pro-inflammatory factors are released during the influenza a virus (IAV)-driven pulmonary immunopathogenesis. PLoS Pathog 2020;16:e1008334. 10.1371/journal.ppat.1008334. 32101596 PMC7062283

[33] Rowe T, Leon AJ, Crevar CJ. et al. Modeling host responses in ferrets during a/California/07/2009 influenza infection. Virology 2010;401:257–65. 10.1016/j.virol.2010.02.020. 20334888 PMC2862141

[34] Di YP, Oslund KL, Zhou X. et al. Synergistic up-regulation of CXCL10 by virus and IFN γ in human airway epithelial cells. PloS One 2014;9(7):e100978. 10.1371/journal.pone.0100978. PMC4102466 25033426

[35] Ormo M, Cubitt AB, Kallio K. et al. Crystal structure of the Aequorea victoria green fluorescent protein. Science 1996;273:1392–5. 10.1126/science.273.5280.1392. 8703075

[36] Kozakov D, Hall DR, Xia B. et al. The ClusPro web server for protein-protein docking. Nat Protoc 2017;12:255–78. 10.1038/nprot.2016.169. 28079879 PMC5540229

[37] Zhong H, Wang D, Fang L. et al. Ubiquitin-specific proteases 25 negatively regulates virus-induced type I interferon signaling. PloS One 2013;8:e80976. 10.1371/journal.pone.0080976. 24260525 PMC3832446

[38] Arimoto K, Takahashi H, Hishiki T. et al. Negative regulation of the RIG-I signaling by the ubiquitin ligase RNF125. Proc Natl Acad Sci U S A 2007;104:7500–5. 10.1073/pnas.0611551104. 17460044 PMC1863485

[39] Zaas AK, Chen M, Varkey J. et al. Gene expression signatures diagnose influenza and other symptomatic respiratory viral infections in humans. Cell Host Microbe 2009;6:207–17. 10.1016/j.chom.2009.07.006. 19664979 PMC2852511

[40] Steeg PS . Targeting metastasis. Nat Rev Cancer 2016;16:201–18. 10.1038/nrc.2016.25. 27009393 PMC7055530

[41] Greene FL, Page DL, Fleming ID. et al. AJCC Cancer Staging Manual, Springer Science & Business Media, USA, 2002.

[42] Pletscher-Frankild S, Palleja A, Tsafou K. et al. DISEASES: text mining and data integration of disease-gene associations. Methods 2015;74:83–9. 10.1016/j.ymeth.2014.11.020. 25484339

[43] Bendas G, Borsig L. Cancer cell adhesion and metastasis: selectins, integrins, and the inhibitory potential of heparins. Int J Cell Biol 2012;2012:1–10. 10.1155/2012/676731. PMC3296185 22505933

[44] Wang JW, Wang SQ, Wu ZY. et al. L1 cell adhesion molecule high expression is associated with poor prognosis in surgically resected brain metastases from lung adenocarcinoma. Clinics (Sao Paulo) 2022;77:100040. 10.1016/j.clinsp.2022.100040. 35525225 PMC9092252

[45] Yang XH, Goldstein A, Sun Y. et al. Detecting critical transition signals from single-cell transcriptomes to infer lineage-determining transcription factors. Nucleic Acids Res 2022;50:e91. 10.1093/nar/gkac452. 35640613 PMC9458468

[46] Zitnik M, Li MM, Wells A. et al. Current and future directions in network biology. arXiv Preprint 2024; arXiv:2309.08478. 10.1093/bioadv/vbae099 PMC11321866 39143982

[47] Li L, Dai H, Fang Z. et al. c-CSN: single-cell RNA sequencing data analysis by conditional cell-specific network. Genomics Proteomics Bioinformatics 2021;19:319–29. 10.1016/j.gpb.2020.05.005. 33684532 PMC8602759

[48] Huang Y, Chang X, Zhang Y. et al. Disease characterization using a partial correlation-based sample-specific network. Brief Bioinform 2021;22:22. 10.1093/bib/bbaa062. 32422654

[49] Dai H, Li L, Zeng T. et al. Cell-specific network constructed by single-cell RNA sequencing data. Nucleic Acids Res 2019;47:e62. 10.1093/nar/gkz172. 30864667 PMC6582408

