# Supplementary material for: Detecting tipping points of complex diseases by network information entropy
Source: Brief Bioinform. 2024 Jul 3;25(4):bbae311. doi: 10.1093/bib/bbae311 (PMC11221888; doi:10.1093/bib/bbae311)
Supplement: Supplementary_materials_final_bbae311 [file supplementary_materials_final_bbae311.pdf]

Supplementary Materials for:

Detecting Tipping Points of Complex Diseases by Network Information Entropy

Chengshang Lyu[1,2], Lingxi Chen[2], Xiaoping Liu[1,*]

[1] Key Laboratory of Systems Health Science of Zhejiang Province, School of Life Science, Hangzhou Institute for Advanced Study, University of Chinese Academy of Sciences, Hangzhou, 310024, China

[2] Department of Biomedical Sciences, City University of Hong Kong, 31 To Yuen Street, Kowloon Tong, Kowloon, Hong Kong 999077, China

[*] To whom correspondence should be addressed. Xiaoping Liu, E-mail: xpliu@ucas.ac.cn

include:

Supplementary Figures

Figure S1. Identification of the pre-disease states of symptomatic subjects infected with H3N2 (GSE52428) using the NIEE algorithm.

Figure S2. Network structure graph and enrichment analysis circle graph of the key local network of Influenza A H3N2 (GSE52428).

Figure S3. Identification of the pre-disease states of symptomatic subjects infected with HRV (GSE17156) using the NIEE algorithm.

Figure S4. In-depth analysis of 24 edges in the key local network of acute lung injury dataset.

Figure S5. In-depth analysis of the key local network of destructive insulitis and hyperglycemia of NOD mice.

Figure S6. Identification of the early-warning signals of H3N2 infected subjects using the NIEE algorithm with the background network integrated String PPI, KEGG, WikiPathways, and Reactome.

Figure S7. Identification of the early-warning signals of H3N2 infected subjects using the NIEE algorithm with Maximal Information Coefficient.

Figure S8. Identification of the early-warning signals of H3N2 infected subjects using the NIEE algorithm with the weight of protein sequence length.

Figure S9. Results of BioTIP Algorithm (cut-off=0.01) on TCGA-LUAD Dataset.

Figure S10. Results of BioTIP Algorithm (cut-off=0.05) on TCGA-LUAD Dataset.

Supplementary Tables

Table S1. The key local network of Influenza A H3N2 GSE30550 datasets.

Table S2. GSE30550 Enrichment analysis chart.

Table S3. The key local network of Influenza A H3N2 GSE52428 datasets.

Table S4. Influenza A H3N2 GSE52428 Enrichment analysis chart.

Table S5. The key local network of HRV GSE17156 datasets.

Table S6. HRV GSE17156 Enrichment analysis chart.

Table S7. The NIEE key local network for GSE2565.

Table S8. The distribution table of the number of patients in each stage of the TCGA-LUAD dataset.

Table S9. The NIEE key network for TCGA-LUAD.

Table S10. TCGA-LUAD Enrichment analysis chart.

Table S11. The NIEE key network for destructive insulitis and hyperglycemia of NOD mice.

Table S12. The key local network of Influenza A H3N2 GSE30550 datasets with the background network integrated String PPI, KEGG, WikiPathways, and Reactome.

Table S13. The key local network of Influenza A H3N2 GSE30550 datasets with Maximal Information Coefficient.

Table S14. The key local network of Influenza A H3N2 GSE30550 datasets with weight of protein sequence length.

Table S15. GSE30550 Enrichment analysis chart with weight of protein sequence length.

Supplementary Notes

Note S1. Analysis of Influenza A H3N2 GSE52428 datasets.

Note S2. Analysis of HRV GSE17156 datasets.

Note S3. The application of NIEE in acute lung injury.

Note S4. Further analysis of genes in the key local network of LUAD.

Note S5. Further exploration - Application of NIEE in destructive insulitis and hyperglycemia of NOD mice with incomplete information.

Note S6. Further exploration - The impact of NIEE on disease early-warning signals in integrated background networks: A case study of GSE30550.

Note S7. Further exploration - The impact of NIEE on disease early-warning signals in different correlation coefficients: A case study of GSE30550.

Note S8. Further exploration - The impact of the genes' weights in NIEE on disease early-warning signals: A case study of GSE30550.

Note S9. Further comparison - The effect of BioTIP and NIEE on disease early-warning signals: A case study of TCGA-LUAD.

Supplementary Figures

Figure S1

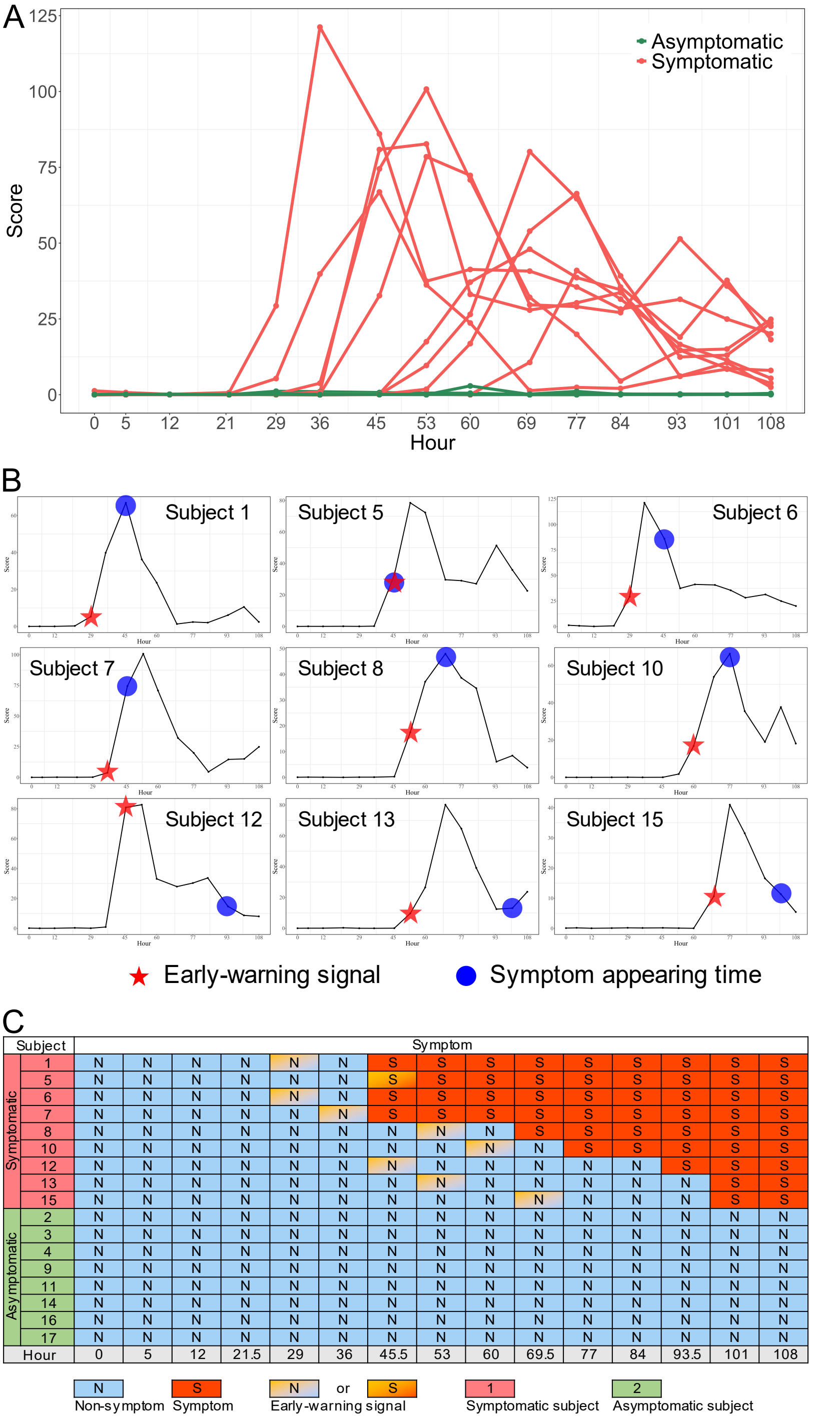

Figure S1. Identification of the pre-disease states of symptomatic subjects infected with H3N2 (GSE52428) using the NIEE algorithm. (A) Line graph of NIEE scores for all subjects, with red indicating scores of symptomatic subjects and green representing scores of asymptomatic subjects. (B) Specific NIEE score line graphs for the nine symptomatic subjects. The red pentagrams signify the moment when the subject's early-warning signal appeared, indicating the NIEE-determined critical pre-disease state. The blue circles represent the moment when the subjects showed clinical symptoms of influenza. (C) Biological temporal table of NIEE personalized early-warning signals and clinical diagnoses for all subjects.

Figure S2

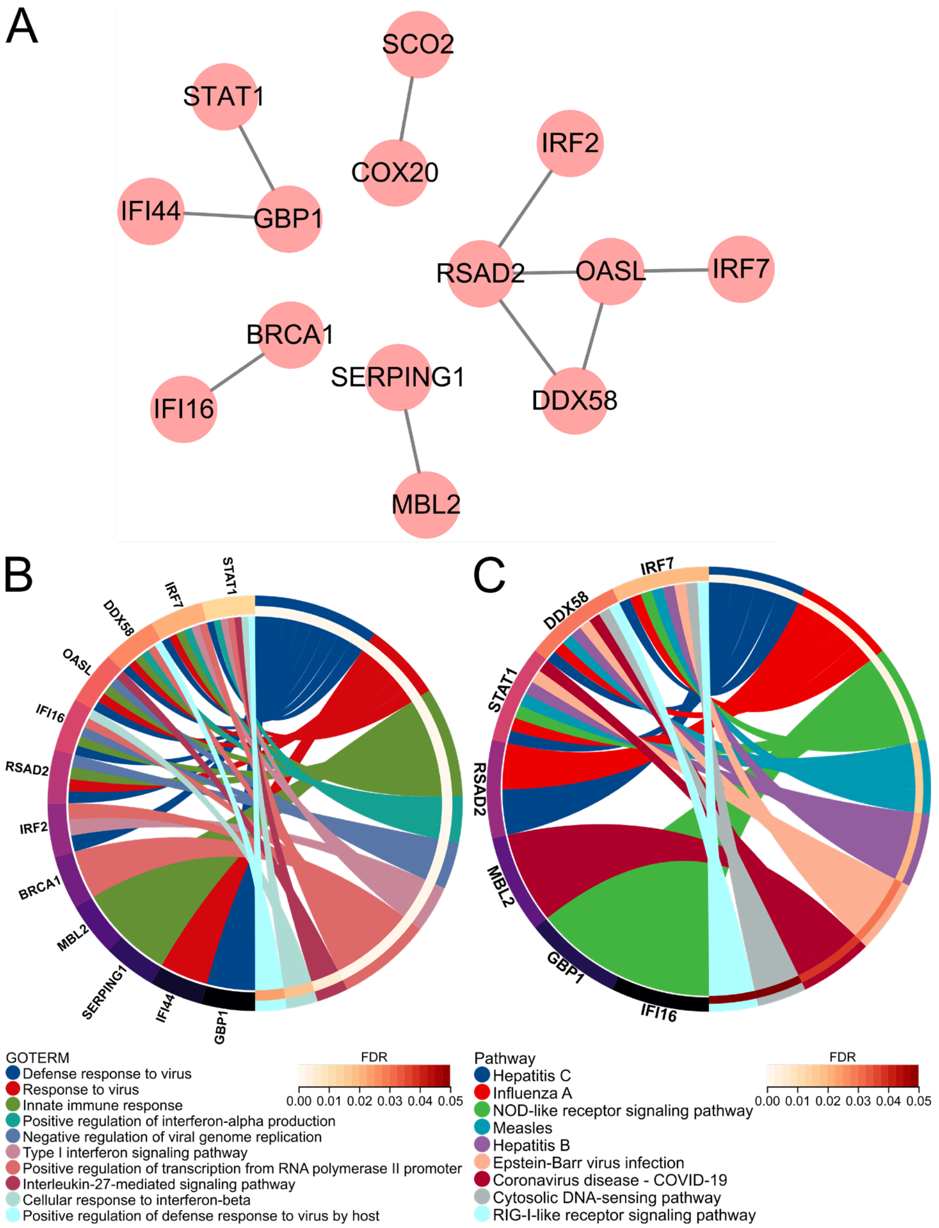

Figure S2. Network structure graph and enrichment analysis circle graph of the key local network of Influenza A H3N2 (GSE52428). (A) The network structure graph for the self-forming network is constituted of all edges in the key local network. (B) Biological process enrichment of all genes in the key local network in GO Process. (C) Pathway enrichment of all genes in the key local network in the KEGG Pathway.

Figure S3

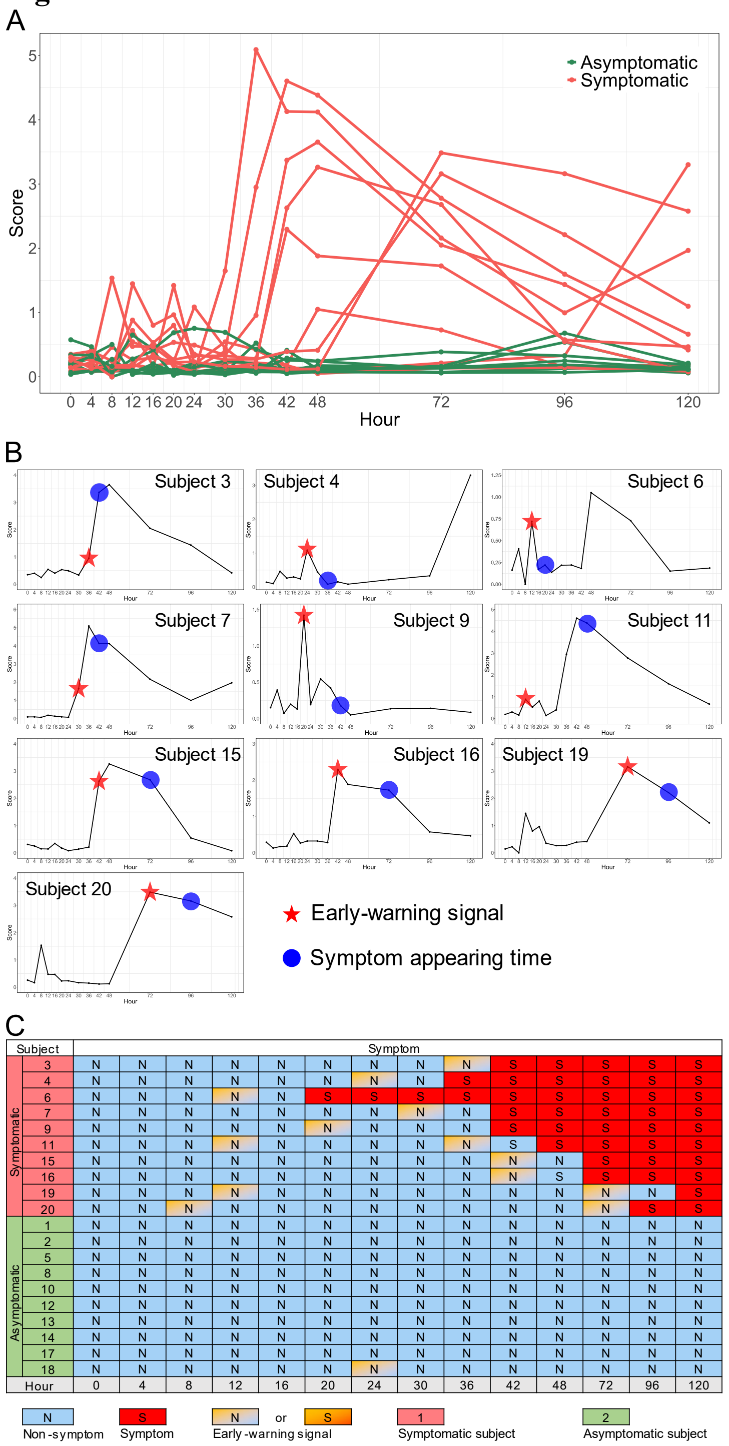

Figure S3. Identification of the pre-disease states of symptomatic subjects infected with HRV (GSE17156) using the NIEE algorithm. (A) Line graph of NIEE scores for all subjects, with red indicating symptomatic subjects' scores and green representing asymptomatic subjects' scores. (B) Specific NIEE score line graphs for the nine symptomatic subjects. The red pentagrams signify the moment when the subject's early-warning signal appeared, indicating the NIEE-determined critical pre-disease state. The blue circles represent the moment when the subjects showed clinical symptoms of HRV. (C) Biological temporal table of NIEE personalized early-warning signals and clinical diagnoses for all subjects.

Figure S4

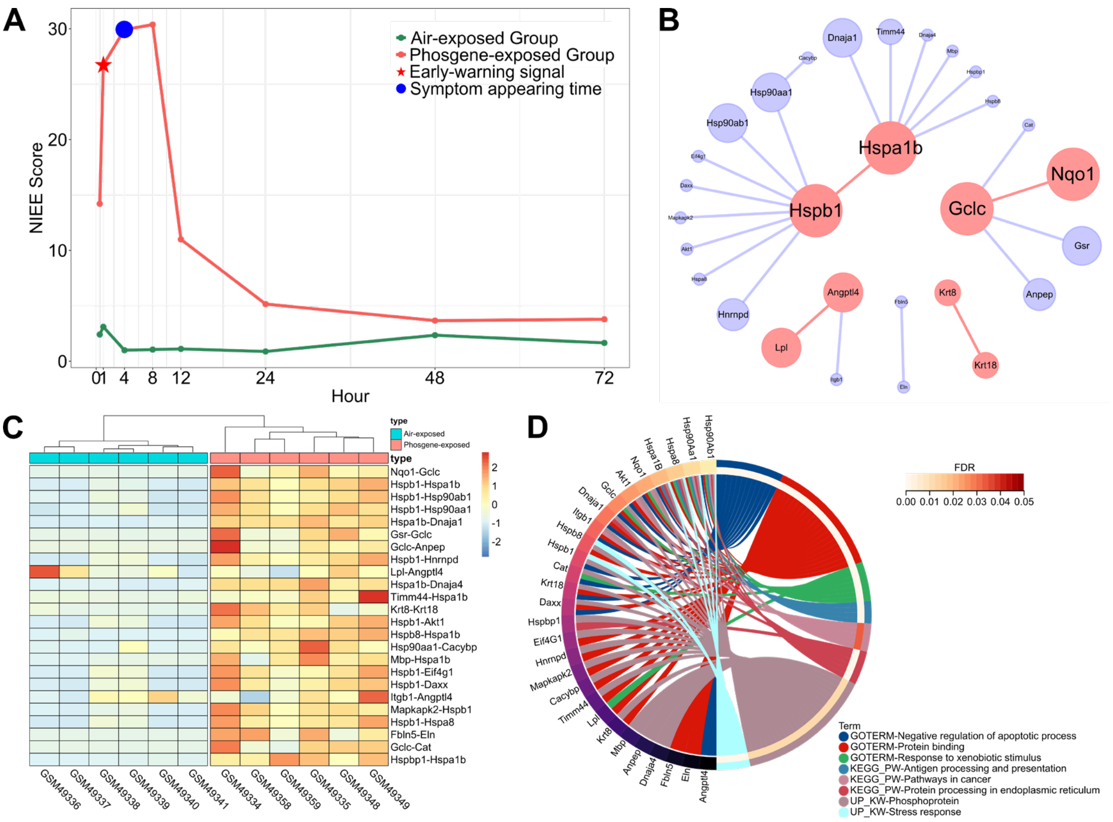

Figure S4. In-depth analysis of 24 edges in the key local network of acute lung injury dataset. (A) The NIEE scores for acute lung injury by phosgene exposure. The green line represents the air-exposed group, while the red line represents the phosgene-exposed group. The NIEE score indicates the early-warning signal and critical point around 1 hour after phosgene exposure. (B) Network structure graph illustrating the self-forming network composed of all edges in the key local network. (C) Heatmap of NIEE scores corresponding to the key local network of each experimental sample at the tipping point (1 hour). (D) Pathway and function enrichment analysis of genes in the key local network.

Figure S5

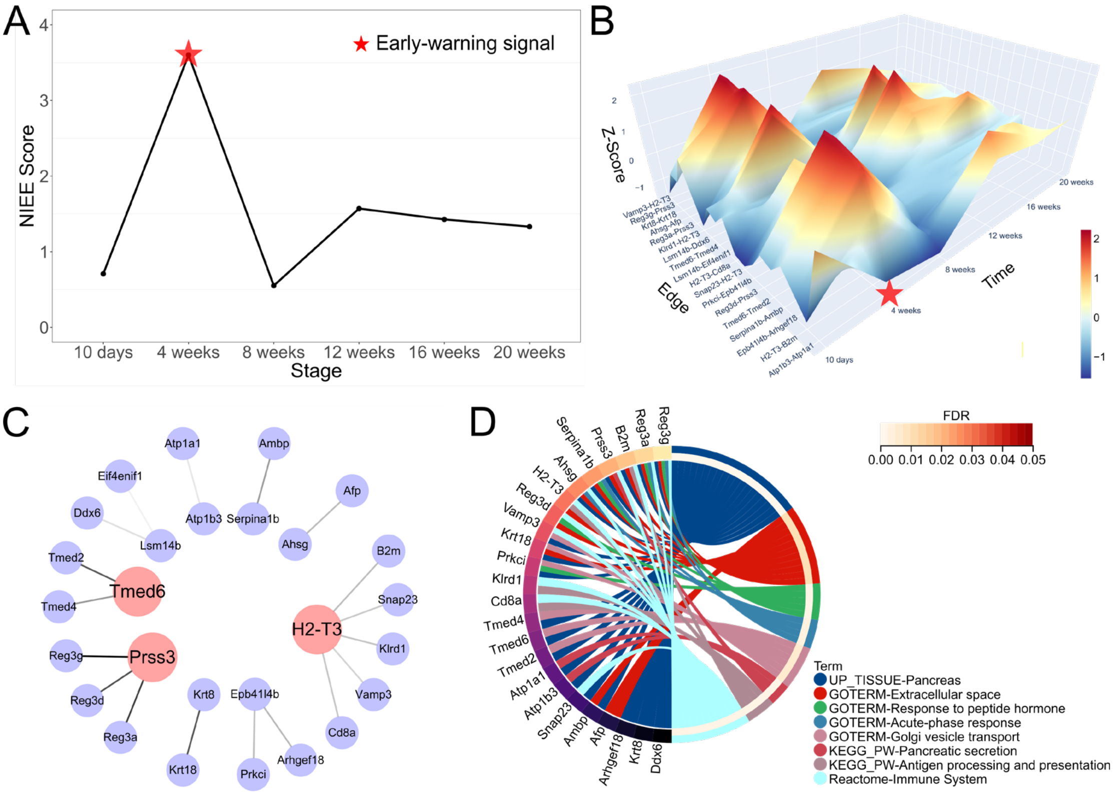

Figure S5. In-depth analysis of the key local network of destructive insulitis and hyperglycemia of NOD mice. (A) NIEE scores line graphs for the key local network, indicating the early-warning signal around four weeks. (B) Landscape graph of z-transformed NIEE scores for the key local network. (C) The network structure graph for the self-forming network constituted of all edges in the key local network. (D) Pathway and function enrichment of genes in the key local network.

Figure S6

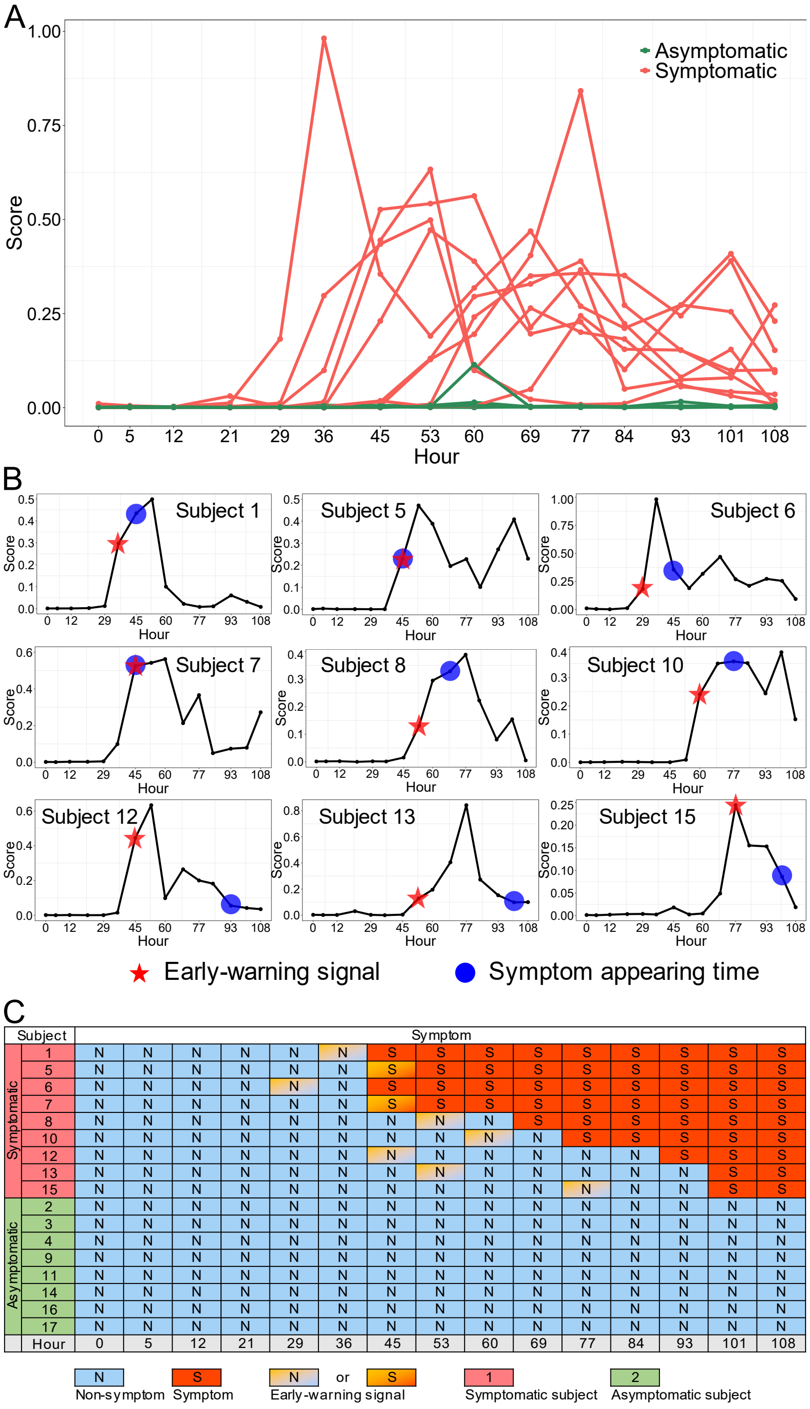

Figure S6. Identification of the early-warning signals of H3N2 infected subjects using the NIEE algorithm with the background network integrated String PPI, KEGG, WikiPathways, and Reactome. (A) Line graph of NIEE scores for all subjects, with red indicating scores of symptomatic subjects and green representing scores of asymptomatic subjects. (B) Specific NIEE score line graphs for the nine symptomatic subjects. The red pentagrams signify the moment when the subject's early-warning signal appeared, indicating the NIEE-determined critical pre-disease state. The blue circles represent the moment when the subjects showed clinical symptoms of influenza. (C) Biological temporal table of NIEE personalized early-warning signals and clinical diagnoses for all subjects.

Figure S7

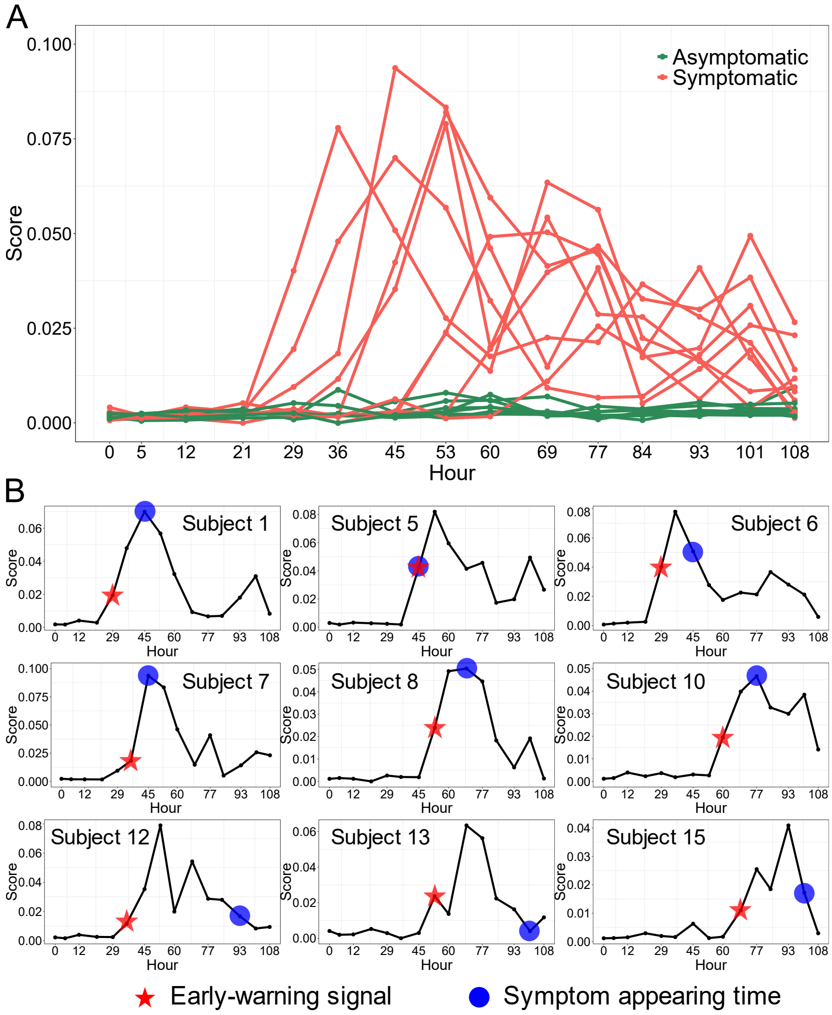

C

Subject		0	5	12	21	29	36	45	53	60	69	77	84	93	101	108
Symptomatic	1	N	N	N	N	N	N	S	S	S	S	S	S	S	S	S
	5	N	N	N	N	N	N	S	S	S	S	S	S	S	S	S
	6	N	N	N	N	N	N	S	S	S	S	S	S	S	S	S
	7	N	N	N	N	N	N	S	S	S	S	S	S	S	S	S
	8	N	N	N	N	N	N	N	N	N	S	S	S	S	S	S
	10	N	N	N	N	N	N	N	N	N	N	S	S	S	S	S
	12	N	N	N	N	N	N	N	N	N	N	N	N	S	S	S
	13	N	N	N	N	N	N	N	N	N	N	N	N	N	S	S
	15	N	N	N	N	N	N	N	N	N	N	N	N	N	S	S
Asymptomatic	2	N	N	N	N	N	N	N	N	N	N	N	N	N	N	N
	3	N	N	N	N	N	N	N	N	N	N	N	N	N	N	N
	4	N	N	N	N	N	N	N	N	N	N	N	N	N	N	N
	9	N	N	N	N	N	N	N	N	N	N	N	N	N	N	N
	11	N	N	N	N	N	N	N	N	N	N	N	N	N	N	N
	14	N	N	N	N	N	N	N	N	N	N	N	N	N	N	N
	16	N	N	N	N	N	N	N	N	N	N	N	N	N	N	N
	17	N	N	N	N	N	N	N	N	N	N	N	N	N	N	N
	Hour	0	5	12	21	29	36	45	53	60	69	77	84	93	101	108

N Non-symptom; S Symptom; N or S Early-warning signal; 1 Symptomatic subject; 2 Asymptomatic subject

Figure S7. Identification of the early-warning signals of H3N2 infected subjects using the NIEE algorithm with Maximal Information Coefficient. (A) Line graph of NIEE scores for all subjects, with red indicating scores of symptomatic subjects and green representing scores of asymptomatic subjects. (B) Specific NIEE score line graphs for the nine symptomatic subjects. The red pentagrams signify the moment when the subject's early-warning signal appeared, indicating the NIEE-determined critical pre-disease state. The blue circles represent the moment when the subjects showed clinical symptoms of influenza. (C) Biological temporal table of NIEE personalized early-warning signals and clinical diagnoses for all subjects.

Figure S8

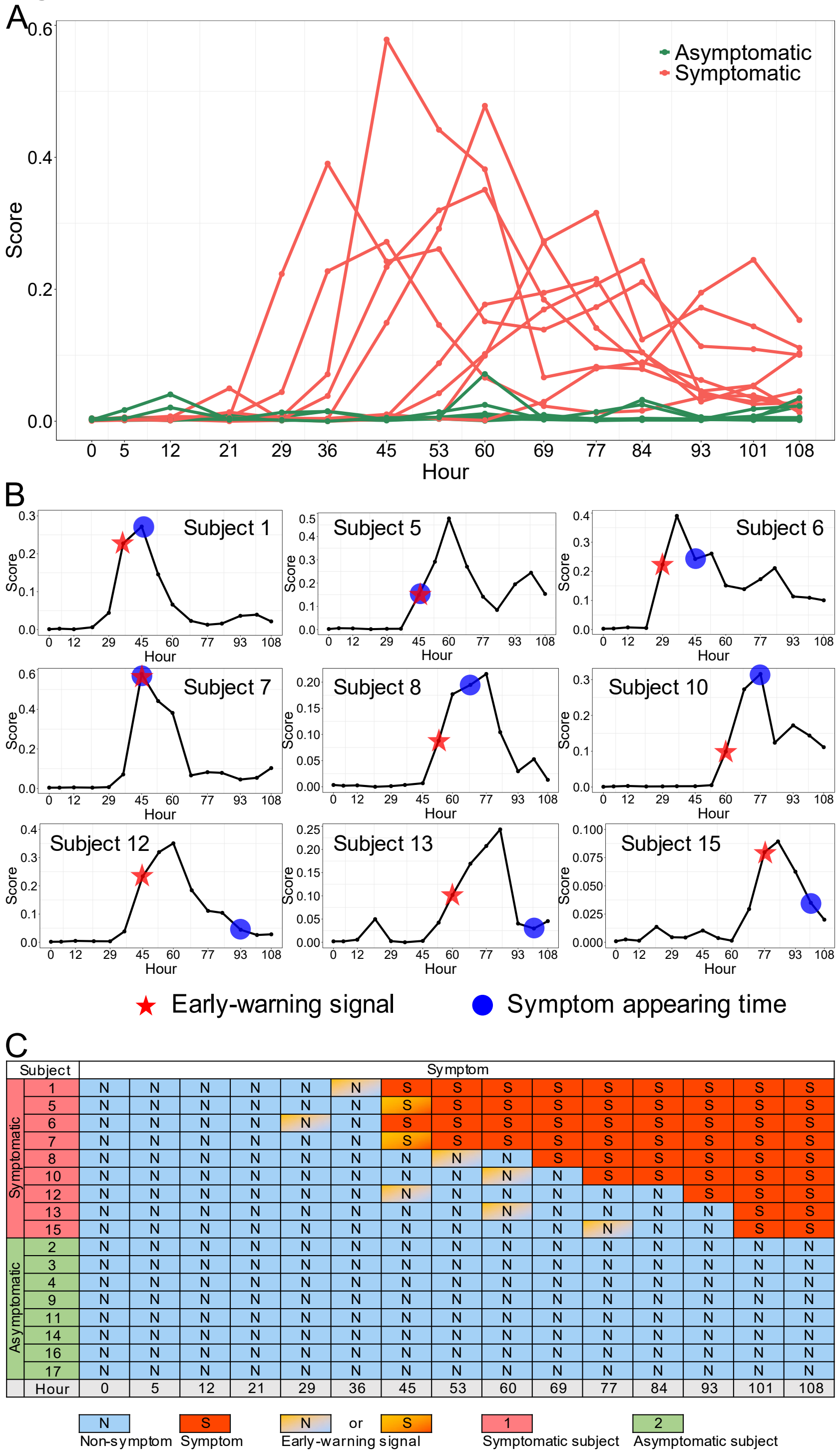

Figure S8. Identification of the early-warning signals of H3N2 infected subjects using the NIEE algorithm with the weight of protein sequence length. (A) Line graph of NIEE scores for all subjects, with red indicating scores of symptomatic subjects and green representing scores of asymptomatic subjects. (B) Specific NIEE score line graphs for the nine symptomatic subjects. The red pentagrams signify the moment when the subject's early-warning signal appeared, indicating the NIEE-determined critical pre-disease state. The blue circles represent the moment when the subjects showed clinical symptoms of influenza. (C) Biological temporal table of NIEE personalized early-warning signals and clinical diagnoses for all subjects.

Figure S9

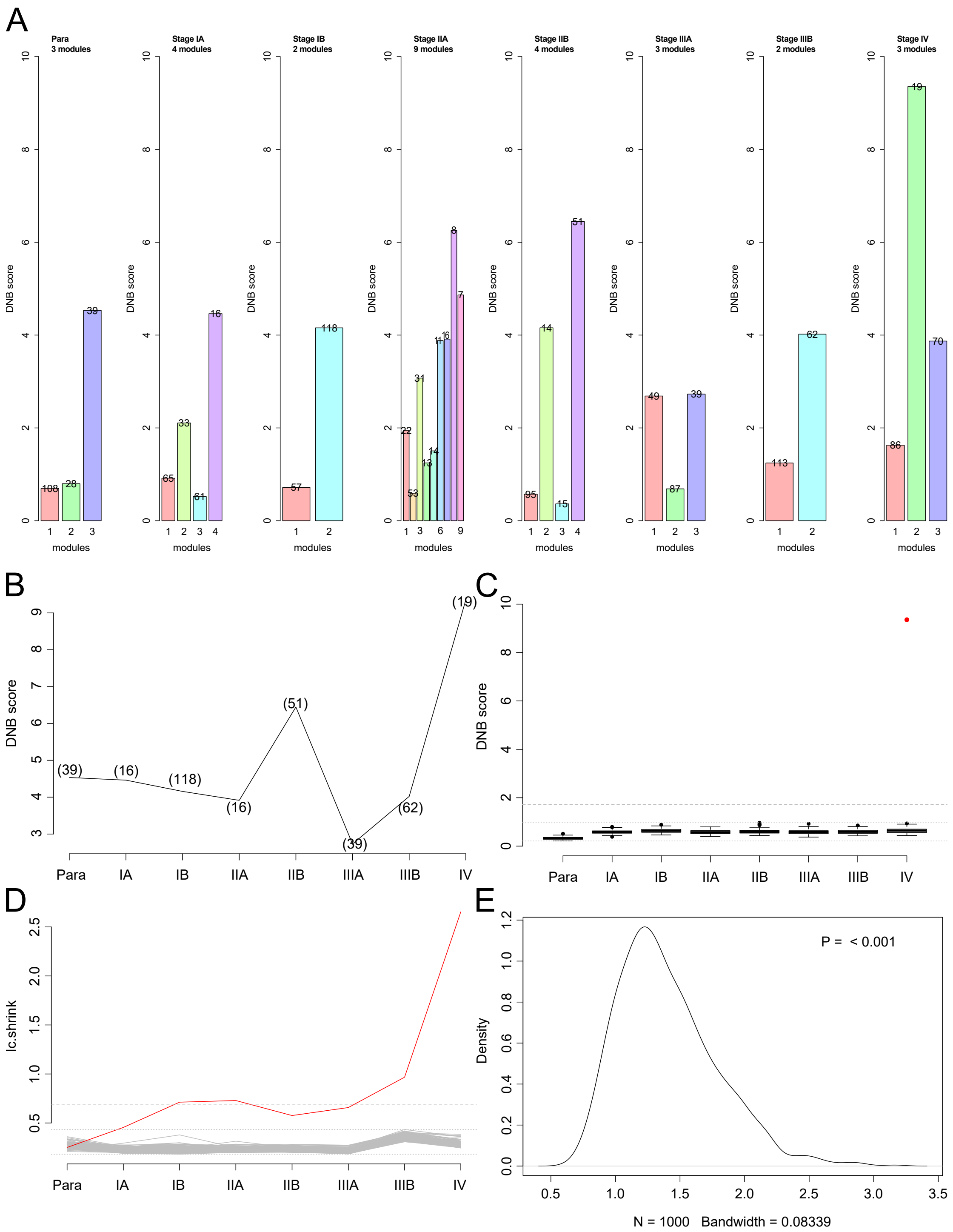

Figure S9. Results of BioTIP Algorithm (cut-off=0.01) on TCGA-LUAD Dataset. (A) MCI scores for modules at each stage. (B) The highest MCI scores at each stage. (C) MCI simulation scores. (D) Ic simulation scores. (E) Density distribution of random Ic scores.

Figure S10

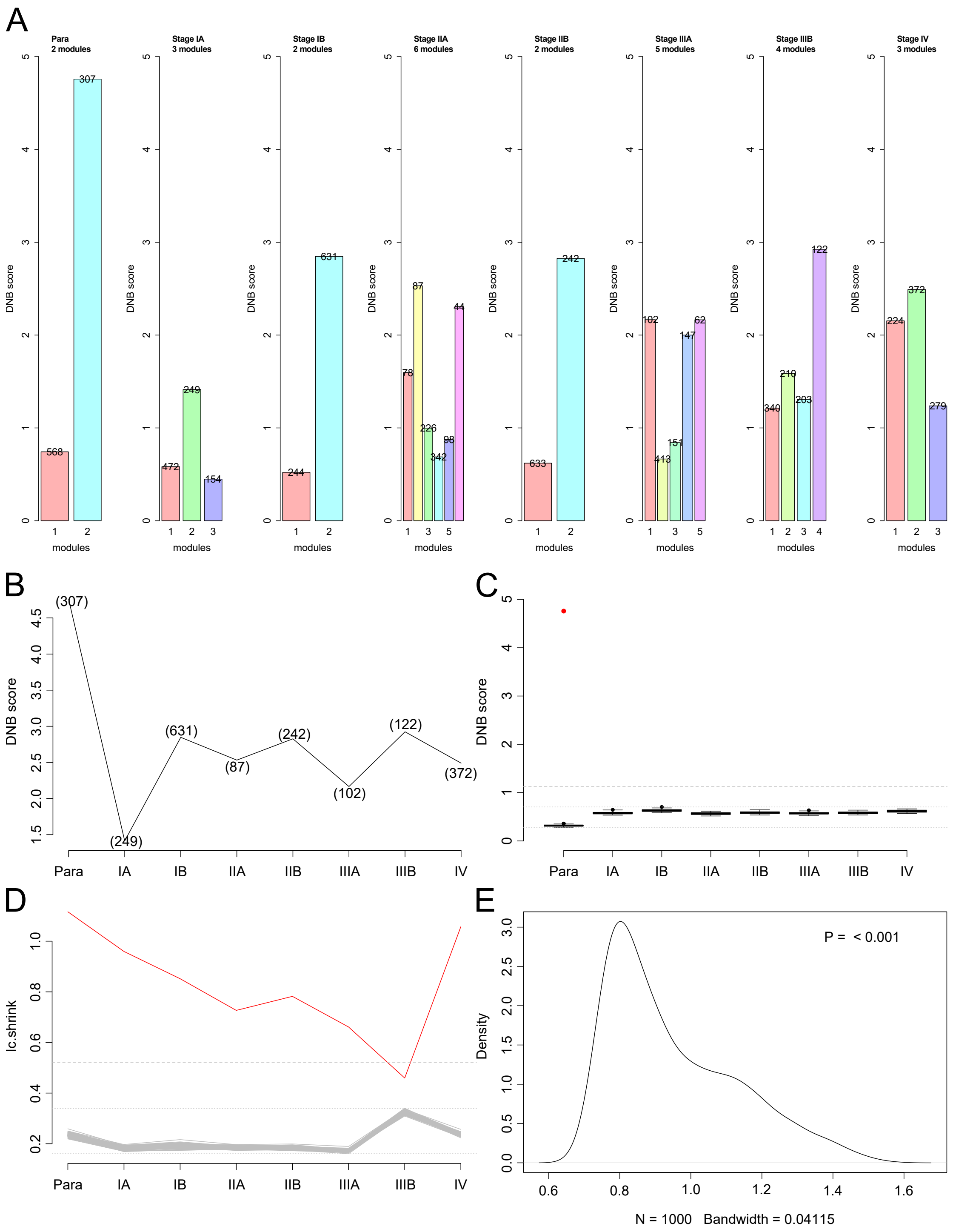

Figure S10. Results of BioTIP Algorithm (cut-off=0.05) on TCGA-LUAD Dataset. (A) MCI scores for modules at each stage. (B) The highest MCI scores at each stage. (C) MCI simulation scores. (D) Ic simulation scores. (E) Density distribution of random Ic scores.

Supplementary Tables

Table S1

The key local network of Influenza A H3N2 GSE30550 datasets.

**Edge**	**Gene1**	**Gene2**
1	RNF125	DDX58
2	CCR1	CCL8
3	CXCL10	CCL8
4	XPNPEP2	LAP3
5	IFIT3	GBP1
6	IFI44	GBP1
7	IFI16	CASP1
8	MICB	KLRK1
9	XCR1	CCL8
10	CCR5	CCL8
11	CCR3	CCL8
12	CCRL2	CCL19
13	USP25	DDX58

Table S2
GSE30550 Enrichment analysis chart.

Type	ID	Enriched Function	Count	Genes	FDR
KEGG Pathway	hsa04061	Viral protein interaction with cytokine and cytokine receptor	7	CCR1, CXCL10, CCL8, XCR1, CCL19, CCR5, CCR3	2.12E-09
	hsa04062	Chemokine signaling pathway	7	CCR1, CXCL10, CCL8, XCR1, CCL19, CCR5, CCR3	9.02E-08
	hsa04060	Cytokine-cytokine receptor interaction	7	CCR1, CXCL10, CCL8, XCR1, CCL19, CCR5, CCR3	9.96E-07
	hsa04622	RIG-I-like receptor signaling pathway	3	CXCL10, RNF125, RIGI	0.0031
	hsa05164	Influenza A	3	CXCL10, CASP1, RIGI	0.0289
Gene Ontology Biological Process	GO:0006955	Immune response	17	CCR1, CCL8, XCR1, CCRL2, IFI44, CCL19, CCR5, CCR3, MICB	1.83E-13
	GO:0070098	Chemokine-mediated signaling pathway	8	CCR1, CXCL10, CCL8, XCR1, CCRL2, CCL19, CCR5, CCR3	9.51E-11
	GO:0006952	Defense response	14	KLRK1, CCR5, CCR1, CXCL10, CCL19, XCR1, CCRL2, IFI16, GBP1, IFIT3, DDX58, CCL8, CASP1, CCR3	4.25E-09
	GO:0009615	Response to virus	8	CXCL10, CCL19, IFI16, GBP1, IFI44, IFIT3, DDX58, CCL8, CASP1	4.63E-08
	GO:0006954	Inflammatory response	9	CXCL10, CCL19, IFI16, GBP1, IFI44, IFIT3, DDX58, CCL8, CASP1	4.63E-08
Uniport Annotated Keywords	KW-0391	Immunity	7	RNF125, KLRK1, MICB, IFI16, GBP1, IFIT3, DDX58	0.00045
	KW-0395	Inflammatory response	4	CXCL10, CCL19, IFI16, CCL8	0.0073
	KW-0145	Chemotaxis	3	CXCL10, CCL19, CCL8	0.0234
	KW-0051	Antiviral defense	3	GBP1, IFIT3, DDX58	0.0395
	KW-0399	Innate immunity	4	KLRK1, IFI16, IFIT3, DDX58	0.039

Table S3

The key local network of Influenza A H3N2 GSE52428 datasets.

Edge	Gene1	Gene2
1	IFI44	GBP1
2	STAT1	GBP1
3	SCO2	COX20
4	OASL	DDX58
5	SERPING1	MBL2
6	IFI16	BRCA1
7	RSAD2	OASL
8	RSAD2	IRF2
9	OASL	IRF7
10	RSAD2	DDX58

Table S4
Influenza A H3N2 GSE52428 Enrichment analysis chart.

Type	ID	Enriched Function	count	Genes	False Discovery Rate
KEGG Pathway	hsa04622	RIG-I-like receptor signaling pathway	2	DDX58, IRF7	0.0475
	hsa05164	Influenza A	4	STAT1, DDX58, RSAD2, IRF7	0.0014
	hsa04621	NOD-like receptor signaling pathway	3	STAT1, GBP1, IRF7	0.0159
Gene Ontology Biological Process	GO:0039528	Cytoplasmic pattern recognition receptor signaling pathway in response to virus	2	DDX58, IRF7	0.015
	GO:0032727	Positive regulation of interferon-alpha production	3	STAT1, DDX58, IRF7	0.00066
	GO:0045071	Negative regulation of viral genome replication	3	OASL, IFI16, RSAD2	0.0051
	GO:0048525	Negative regulation of viral process	5	OASL, STAT1, IFI16, MBL2, RSAD2	6.72E-06
	GO:0051607	Defense response to virus	9	OASL, STAT1, IFI16, GBP1, MBL2, DDX58, RSAD2, IRF2, IRF7	1.61E-10
	GO:0009615	Response to virus	10	OASL, STAT1, IFI16, GBP1, MBL2, IFI44, DDX58, RSAD2, IRF2, IRF7	6.33E-11
	GO:0006955	Immune	10	OASL, SERPING1, STAT1,	2.33E-06

		response		IFI16, GBP1, IFI44, MBL2, DDX58, RSAD2, IRF7	
Uniport Annotated Keywords	KW-0180	Complement pathway	2	SERPING1, MBL2	0.0308
	KW-0051	Antiviral defense	6	OASL, STAT1, GBP1, DDX58, RSAD2, IRF7	1.63e-07
	KW-0399	Innate immunity	7	OASL, SERPING1, IFI16, MBL2, DDX58, RSAD2, IRF7	2.83e-07
	KW-0391	Immunity	8	OASL, SERPING1, IFI16, GBP1, MBL2, DDX58, RSAD2, IRF7	2.83e-07

Table S5
The key local network of HRV GSE17156 datasets.

Edge	Gene1	Gene2
1	S100P	CACYBP
2	LORICRIN	KRT1
3	HNMT	AOC1
4	KRT1	FLG
5	IFI44L	IFI35
6	OAS1	IFI44L
7	RSAD2	IFIT2
8	SIPA1L1	RAP1A
9	RTP4	IFI44L
10	RSAD2	IFI44
11	RSAD2	OAS3
12	SIGLEC1	CD163
13	IFI44L	DDX60
14	OAS3	IFI44L
15	RTP4	IFI44
16	SIGLEC1	GP5
17	DFFA	CASP3
18	IFI44L	HERC5
19	XAF1	IFI44L
20	IFIT1	IFI44L
21	IFI44L	HERC6
22	IFIT2	IFI44L

Table S6
HRV GSE17156 Enrichment analysis chart.

Type	ID	Enriched Function	count	Genes	False Discovery Rate
GO Process	GO:0009607	Response to biotic stimulus	16	OAS3, KRT1, RTP4, HERC6, HERC5, HNMT, CASP3, XAF1, IFI44, IFI44L, IFIT1, IFIT2, RSAD2, DDX60, OAS1, IFI35	1.14E-08
	GO:0009615	Response to virus	10	OAS3, RTP4, HERC5, IFI44, IFI44L, IFIT1, IFIT2, RSAD2, DDX60, OAS1	2.90E-08
	GO:0051607	Defense response to virus	9	OAS3, RTP4, HERC5, IFI44L, IFIT1, IFIT2, RSAD2, DDX60, OAS1	2.93E-08
	GO:0051707	Response to other organism	15	OAS3, KRT1, RTP4, HERC6, HERC5, CASP3, XAF1, IFI44, IFI44L, IFIT1, IFIT2, RSAD2, DDX60, OAS1, IFI35	2.93E-08
	GO:0060337	Type I interferon signaling pathway	7	OAS3, XAF1, IFIT1, IFIT2, RSAD2, OAS1, IFI35	2.93E-08
	GO:0002252	Immune effector process	13	OAS3, KRT1, RTP4, HERC5, S100P, RAP1A, IFI44L, IFIT1, IFIT2, RSAD2, DDX60, OAS1, AOC1	1.60E-07
	GO:0002376	Immune system process	18	OAS3, KRT1, RTP4, HERC6, HERC5, S100P, CASP3, XAF1, RAP1A, IFI44, IFI44L, IFIT1, IFIT2, RSAD2, DDX60, OAS1, IFI35, AOC1	1.60E-07
	GO:0006952	Defense response	14	OAS3, KRT1, RTP4, HERC5, SIGLEC1, CD163, XAF1, IFI44L, IFIT1, IFIT2, RSAD2, DDX60, OAS1, IFI35	3.42E-07

	GO:0006955	Immune response	15	OAS3, KRT1, HERC5, S100P, XAF1, RAP1A, IFI44, IFI44L, IFIT1, IFIT2, RSAD2, DDX60, OAS1, IFI35, AOC1	3.42E-07
	GO:0044419	Interspecies interaction between organisms	16	OAS3, KRT1, RTP4, HERC6, HERC5, CASP3, SIGLEC1, XAF1, IFI44, IFI44L, IFIT1, IFIT2, RSAD2, DDX60, OAS1, IFI35	3.42E-07
	GO:0098542	Defense response to other organism	12	OAS3, KRT1, RTP4, HERC5, XAF1, IFI44L, IFIT1, IFIT2, RSAD2, DDX60, OAS1, IFI35	8.05E-07
	GO:0009605	Response to external stimulus	16	OAS3, KRT1, RTP4, HERC6, HERC5, CASP3, XAF1, IFI44, IFI44L, IFIT1, IFIT2, RSAD2, DDX60, OAS1, IFI35, AOC1	4.56E-06
	GO:0045087	Innate immune response	10	OAS3, KRT1, HERC5, XAF1, IFIT1, IFIT2, RSAD2, DDX60, OAS1, IFI35	1.48E-05
	GO:0050896	Response to stimulus	24	OAS3, KRT1, RTP4, HERC6, HERC5, HNMT, S100P, CASP3, SIGLEC1, CD163, XAF1, CACYBP, RAP1A, IFI44, IFI44L, IFIT1, IFIT2, RSAD2, DDX60, GP5, OAS1, IFI35, AOC1, SIPA1L1	0.00026
	GO:0034097	Response to cytokine	10	OAS3, HNMT, CASP3, XAF1, CACYBP, IFIT1, IFIT2, RSAD2, OAS1, IFI35	0.00078
	GO:0045071	Negative regulation of viral genome replication	4	OAS3, IFIT1, RSAD2, OAS1	0.0012
	GO:0019221	Cytokine-mediated signaling pathway	8	OAS3, CASP3, XAF1, IFIT1, IFIT2, RSAD2, OAS1, IFI35	0.0016

	GO:0071345	Cellular response to cytokine stimulus	9	OAS3, CASP3, XAF1, CACYBP, IFIT1, IFIT2, RSAD2, OAS1, IFI35	0.0031
KEGG	hsa05160	Hepatitis C	5	OAS3, CASP3, IFIT1, RSAD2, OAS1	0.00083
	hsa05164	Influenza A	4	OAS3, CASP3, RSAD2, OAS1	0.0135
Reactome	HSA-909733	Interferon alpha/beta signaling	7	OAS3, XAF1, IFIT1, IFIT2, RSAD2, OAS1, IFI35	1.80E-08
	HSA-913531	Interferon Signaling	8	OAS3, HERC5, XAF1, IFIT1, IFIT2, RSAD2, OAS1, IFI35	2.45E-07
	HSA-168256	Immune System	15	OAS3, KRT1, HERC6, HERC5, S100P, CASP3, SIGLEC1, XAF1, RAP1A, IFIT1, IFIT2, RSAD2, OAS1, IFI35, AOC1	4.04E-06
	HSA-1280215	Cytokine Signaling in Immune system	9	OAS3, HERC5, CASP3, XAF1, IFIT1, IFIT2, RSAD2, OAS1, IFI35	0.00011
	HSA-1169410	Antiviral mechanism by IFN-stimulated genes	4	OAS3, HERC5, IFIT1, OAS1	0.0023
	HSA-8983711	OAS antiviral response	2	OAS3, OAS1	0.0361
Uniprot Keywords	KW-0051	Antiviral defense	8	OAS3, HERC5, IFI44L, IFIT1, IFIT2, RSAD2, DDX60, OAS1	5.54E-09
	KW-0399	Innate immunity	7	OAS3, HERC5, IFIT1, IFIT2, RSAD2, DDX60, OAS1	8.27E-05
	KW-0963	Cytoplasm	18	OAS3, HERC6, HERC5, HNMT, S100P, CASP3, XAF1, CACYBP, LOR, RAP1A, IFI44, IFI44L, IFIT1, IFIT2, DFFA, DDX60, OAS1, SIPA1L1	0.0016

Table S7
The NIEE key local network for GSE2565.

Edge	Gene1	Gene2
1	Nqo1	Gclc
2	Hspb1	Hspa1b
3	Hspb1	Hsp90ab1
4	Hspb1	Hsp90aa1
5	Hspa1b	Dnaja1
6	Gsr	Gclc
7	Gclc	Anpep
8	Hspb1	Hnrnpd
9	Lpl	Angptl4
10	Hspa1b	Dnaja4
11	Timm44	Hspa1b
12	Krt8	Krt18
13	Hspb1	Akt1
14	Hspb8	Hspa1b
15	Hsp90aa1	Cacybp
16	Mbp	Hspa1b
17	Hspb1	Eif4g1
18	Hspb1	Daxx
19	Itgb1	Angptl4
20	Mapkapk2	Hspb1
21	Hspb1	Hspa8
22	Fbln5	Eln
23	Gclc	Cat
24	Hspbp1	Hspa1b

Table S8
The distribution table of the number of patients in each stage of the TCGA-LUAD dataset.

**Stage**	**IA**	**IB**	**IIA**	**IIB**	**IIIA**	**IIIB**	**IV**
Number of samples	134	147	50	71	73	11	26

Table S9
The NIEE key network for TCGA-LUAD.

**Edge**	**Gene1**	**Gene2**
1	S100P	AGER
2	SFTPC	SFTPA2
3	SFTPC	PIGR
4	SFTPC	ADRA2A
5	NEIL3	EXO1
6	FGL1	FGG
7	TRPV2	TRPM8
8	TOP2A	NEIL3
9	SFTPC	NAPSA
10	FGL1	FGB
11	STRA6	RBP4
12	PMEL	MAGEA3
13	TRIM28	MAGEA3
14	SFTPC	SFTPA1
15	SFTPB	NAPSA

Table S10

TCGA-LUAD Enrichment analysis chart.

Type	ID	Enriched Function	count	Genes	False Discovery Rate
Gene Ontology Biological Process	GO:0034116	Positive regulation of heterotypic cell-cell adhesion	3	FGB, FGG, AGER	0.0055
	GO:0007585	Respiratory gaseous exchange by respiratory system	4	SFTPC, SFTPA2, SFTPA1, SFTPB	0.0055
	GO:0032501	Multicellular organismal process	19	TRIM28, NAPSA, ADRA2A, S100P, FGB, SFTPC, TRPM8, FGG, TRPV2, PIGR, EXO1, RBP4, SFTPA2, AGER, FGL1, SFTPA1, TOP2A, STRA6, SFTPB	0.0055
Gene Ontology Cellular Component	GO:0097486	Multivesicular body lumen	3	SFTPB, NAPSA, SFTPC	9.82E-05
	GO:0097208	Alveolar lamellar body	3	SFTPB, NAPSA, SFTPC	9.82E-05
	GO:0005577	Fibrinogen complex	3	FGB, FGL1, FGG	9.82E-05
	GO:0042599	Lamellar body	5	NAPSA, SFTPB, SFTPA2, SFTPC, SFTPA1	5.64E-08
	GO:0005771	Multivesicular body	6	NAPSA, SFTPC, SFTPA2, SFTPA1, PMEL, SFTPB	1.28E-07
Monarch Initiative Human Phenotype	HP:0032975	Abnormal bronchoalveolar fluid protein level	2	SFTPC, SFTPB	0.0289
	HP:0032973	Abnormal bronchoalveolar lavage fluid morphology	3	SFTPC, SFTPA2, SFTPB	0.0032
	HP:0033711	Desquamative interstitial pneumonitis	2	SFTPC, SFTPB	0.0361
	HP:0012223	Splenic rupture	2	FGB, FGG	0.0361

	HP:0025389	Pulmonary interstitial high-resolution computed tomography abnormality	3	SFTPC, SFTPA2, SFTPB	0.0032
Jensen Lab Disease-gene associations	DOID:850	Lung disease	6	NAPSA, SFTPC, SFTPA2, AGER, SFTPA1, SFTPB	0.00018

Table S11

The NIEE key network for destructive insulitis and hyperglycemia of NOD mice.

Edge	Gene1	Gene2
1	Atp1b3	Atp1a1
2	H2-T3	B2m
3	Epb41l4b	Arhgef18
4	Serpina1b	Ambp
5	Tmed6	Tmed2
6	Reg3d	Prss3
7	Prkci	Epb41l4b
8	Snap23	H2-T3
9	H2-T3	Cd8a
10	Lsm14b	Eif4enif1
11	Tmed6	Tmed4
12	Lsm14b	Ddx6
13	Klrd1	H2-T3

Table S12
The key local network of Influenza A H3N2 GSE30550 datasets with the background network integrated String PPI, KEGG, WikiPathways and Reactome.

**Edge**	**Gene1**	**Gene2**
1	CXCL10	CCL8
2	CCR1	CCL8
3	IFIH1	DHX58
4	XPNPEP2	LAP3
5	IFIT3	GBP1
6	MICB	KLRK1
7	TDRD7	TACC1
8	CCR3	CCL8
9	CCRL2	CCL19
10	RNF125	DDX58
11	USP25	DDX58
12	IFI44	GBP1
13	CCR5	CCL8
14	XCR1	CCL8

Table S13

The key local network of Influenza A H3N2 GSE30550 datasets with Maximal Information Coefficient.

**Edge**	**Gene1**	**Gene2**
1	TDRD7	CDK17
2	XPNPEP1	LAP3
3	NAT8	LAP3
4	PRODH	LAP3
5	SRM	SAT1
6	XPNPEP2	LAP3
7	IFI16	DDX41
8	GCH1	ALPG
9	LAP3	DNPEP
10	TRIM5	BTBD1
11	SNTB1	DTNB
12	PYCR3	LAP3
13	SNX2	FNBP1

Table S14
The key local network of Influenza A H3N2 GSE30550 datasets with weight of protein sequence length.

**Edge**	**Gene1**	**Gene2**
1	CXCL10	CCL8
2	IFIT3	GBP1
3	STAT1	GBP1
4	NFIL3	BHLHE41
5	CCR1	CCL8
6	FOXO1	CCNG2
7	IFI16	CASP1
8	TDRD7	CDK17
9	XPNPEP2	LAP3
10	ZBP1	IRF7
11	CCR5	CCL8
12	TLR4	CIRBP
13	IFI44	GBP1

Table S15

GSE30550 Enrichment analysis chart with weight of protein sequence length.

Type	ID	Enriched Function	Count	Genes	FDR
KEGG Pathway	hsa04623	Cytosolic DNA-sensing pathway	5	ZBP1, CXCL10, IFI16, IRF7, CASP1	5.22E-04
	hsa04621	NOD-like receptor signaling pathway	6	IFI16, STAT1, IRF7, CASP1, GBP1, TLR4	5.22E-04
	hsa05164	Influenza A	5	CXCL10, STAT1, IRF7, CASP1, TLR4	0.0050
	hsa04062	Chemokine signaling pathway	5	CCR1, CXCL10, CCL8, STAT1, CCR5	0.0058
	hsa04661	Viral protein interaction with cytokine and cytokine receptor	4	CCR1, CXCL10, CCL8, CCR5	0.0100
	hsa04620	Toll-like receptor signaling pathway	4	CXCL10, STAT1, IRF7, TLR4	0.0104
Gene Ontology Biological Process	GO:0051607	Defense response to virus	7	ZBP1, IFI16, STAT1, IRF7, CASP1, GBP1, IFIT3	4.14E-05
	GO:0071346	Cellular response to interferon-gamma	5	CCL8, STAT1, CASP1, GBP1, TLR4	6.18E-04
	GO:0070098	Chemokine-mediated signaling pathway	4	CCR1, CXCL10, CCL8, CCR5	0.0051
	GO:0006954	Inflammatory response	6	CCR1, CXCL10, CCL8, IFI16, CCR5, TLR4	0.0051
	GO:006935	Chemotaxis	4	CCL8, IRF7, IFI44, IFIT3	0.0122
	GO:0006955	Immune response	5	CCR1, NFIL3, IFI44, CCR5, TLR4	0.0555
Uniport Annotated Keywords	KW-0051	Antiviral defense	5	ZBP1, STAT1, IRF7, GBP1, IFIT3	5.59E-04
	KW-0399	Innate immunity	6	ZBP1, IFI16, IRF7, GBP1, TLR4, IFIT3	0.0019
	KW-0395	Inflammatory response	4	CXCL10, CCL8, IFI16, TLR4	0.0133
	KW-0053	Apoptosis	4	ZBP1, IFI16, CASP1, FOXO1	0.1400
	KW-0945	Host-virus interaction	4	ZBP1, STAT1, IRF7, CCR5	0.2016

Supplementary Notes

Note S1

Analysis of Influenza A H3N2 GSE52428 datasets

Similar to GSE30550, 17 healthy humans were challenged with live H3N2 viruses, and authors obtained subjects' peripheral blood gene expression at 16-time points over 132 hours (hour -24, 0, 5, 12, 21.5, 29, 36, 45.5, 53, 60, 69.5, 77, 84, 93.5, 101, 108). Among these subjects, 9 had flu symptoms (subjects 1, 5, 6, 7, 8, 10, 12, 13, 15), while the rest were asymptomatic (subjects 2, 3, 4, 9, 11, 14, 16, 17). We regarded the samples of all subjects at the -24hour time point as the reference sample group, meaning that these samples were all healthy and were not affected by the virus injection.

Utilizing the NIEE algorithm, we calculated each differential NIEE score. Based on the global differential NIEE score occurrence frequency, a key local network (including ten key edges) was selected from the top 1% differential local NIEE score at every moment of the highest global differential NIEE score among all symptomatic subjects. The sum of the key local network's differential NIEE score served as a quantitative judgment, with our goal being to identify every tipping point predicting the onset of the disease.

The results demonstrate a clear contrast in the key differential local NIEE scores between subjects with and without clinical symptoms (Figure S1A). On the one hand, all the symptomatic subjects have early-warning signals, and 8 of them have NIEE early-warning signals before clinical symptoms appear (Figure S1B and S1C). On the other hand, none of the asymptomatic subjects show early-warning signals. For the H3N2 influenza virus, the key local network identified by the NIEE algorithm can effectively identify samples at the onset of clinical symptoms and provide individualized early-warning signals for everyone with precision. Furthermore, we can use the key local network to predict disease in other samples or patients.

According to the key local networks formed by the ten edges, these edges can spontaneously form multiple small networks (Figure S2A). Also, we put these 14 genes into StringDB with medium confidence $(score \geq 0.4)$(Figure S2B), which shows close connections between genes, and the genes are closely related to immune response, interferon, response to virus, and influenza A (Figure S2C, S2D and Table S4).

Note S2

Analysis of HRV GSE17156 datasets

HRV stands out as one of the primary causative factors for respiratory infections in individuals. Identifying early-warning signals of HRV virus before symptoms appear is of great significance for rapid recovery and control of disease transmission after infection. Hereby, the NIEE method was utilized to calculate the NIEE scores of each HRV subject in the GSE17156 dataset [1] at various times with 22 edges of the key local network (details in Table S5).

As shown in Figure S3A, there was a total of 20 subjects in the experiment. According to the modified Jackson score [2, 3], 10 of them showed obvious symptoms. And the remaining ten individuals did not show any clinical symptoms throughout the entire experimental process.

The specific NIEE scores of symptomatic subjects and the overall situation of all subjects are shown in Figures S3B and S3C. The NIEE scores of all symptomatic subjects rapidly increased in the critical states, and the early-warning signals were all before the onset of clinical symptoms. For the 18th asymptomatic subject, due to its peak NIEE scores exceeding the threshold of the NIEE warning signal at the 24th hour, a false alarm occurred. We speculate that such false positives may be related to the adverse reactions or asymptomatic infections that this asymptomatic subject experienced after being inoculated with the HRV virus.

The enrichment analysis of the key networks is shown in Table S6. Most biological processes and pathways are related to keywords such as immunity and antiviral activity. It is worth noting that in our identified key local network, the subnetworks composed of edges RSAD2-IFIT2, RSAD2-IFI44, and RSAD2-OAS3 highly overlap with the HRV predictive factor RSAD2 and the OAS gene family mentioned in previous research [1, 4, 5].

The successful identification of early-warning signals for HRV has further illustrated that NIEE has good accuracy and effectiveness in predicting the onset of various acute diseases.

Note S3

The application of NIEE in acute lung injury

The study by Duniho et al. investigated the molecular mechanisms underlying phosgene-induced lung injury using RNA data from CD-1 male mice lung tissue [6]. They conducted an experiment wherein they monitored changes in redox enzyme activity and gene expression over a duration of time following a 20-minute exposure to phosgene. A comparative analysis was performed against a control group exposed to ambient air. The lung tissues were gathered from mice exposed to air or phosgene at intervals of 0.5, 1, 4, 8, 12, 24, 48, and 72 hours post-exposure. Given that exposure to phosgene can potentially result in latent, life-threatening pulmonary edema within the span of 1 to 24 hours, followed by irreversible acute lung injury, the researchers employed genomic methodologies to probe into the intricate molecular mechanisms governing phosgene-induced lung injury.

The bronchoalveolar lavage fluid protein level of mice in the phosgene-exposed group increased at 8 hours after exposure. The symptoms of pulmonary edema were increased in the phosgene-exposed group, and the final survival rate was decreased. Besides, various gene expressions involved in the antioxidant response in lung tissue peaked at the 4th or 8th hour after exposure [7].

Due to the irreversible lung damage following the latent period of phosgene exposure, NIEE was utilized to detect early-warning signals and key local networks during the incubation period in order to reduce more harm caused by phosgene exposure. Six sets of RNA-seq data from three naive mice served as reference samples, while experimental mice exposed to phosgene or air were treated as perturbed samples. After that, a background network was constructed based on StringDB with a threshold of 0.85 to calculate the NIEE score of each experimental mouse, and then the mean value of the NIEE scores at each time point was computed. Following this, we determined a key local network by evaluating the frequency of the top 100 edges with the highest NIEE scores in each sample. Specifically, we selected edges that appeared at least 24 times in the 48 phosgene-exposed samples, resulting in a key local network comprised of 24 key edges (Figure S4B and Table S7). Simultaneously, the visualization of the key local network is shown with different sizes and colors based on the NIEE scores of edges and the connectivity of each gene in Figure S4B.

The NIEE scores exhibited a remarkable disparity between the air-exposed group and the phosgene-exposed group, with a significant p-value of 0.0048 (Figure S4A). The NIEE score of the phosgene-exposed group was noticeably higher than that of the air-exposed group at 0.5 hours after gas exposure. Next, the NIEE score rapidly increased in the first hour, indicating the emergence of the early-warning signal, and the system entered the pre-disease state around that moment. Moreover, this early-warning signal observed at 1 hour preceded the appearance of obvious lung damage and the activation of various antioxidant reactions and redox enzymes at the 4-hour mark in the original experiment. To enhance interpretability, we standardized the NIEE scores of each sample at the tipping point and plotted the corresponding heatmap (Figure S4C). In the clustering analysis of the heatmap, clear differentiation between the air-exposed and phosgene-exposed groups was observed. This distinction underscores the sensitivity of NIEE scores in delineating the effects of air and phosgene exposure on the biological system.

Compared with the previous methods proposed by Liu et al. [8, 9], NIEE provides a more advanced early warning for pulmonary edema (1 hour vs. 8 hours). We believe that such earlier

advance warning can lead to earlier and more proactive treatment for potential victims in future practical applications.

In the enrichment analysis for the genes in the key local network (Figure S4D), certain functions were found to be closely associated with keywords such as xenobiotic stimulation, stress response, and antigen processing, indicating that the key local networks may possess the capability to recognize changes in the extracellular environment of mouse lungs. Furthermore, this key local network may serve as early-warning signals for pulmonary edema, as genes in it are linked to biological processes and pathways indicative of responses to environmental stressors and potential threats. This insight reinforces the potential utility of the identified key local network in predicting and responding to pulmonary edema triggered by phosgene exposure.

In the key local network, the prominence of genes in the heat shock protein family is noteworthy and demands our attention. Among them, HSPB1-HSPA1B had a high NIEE score at the tipping point, and HSPB1 is recognized as a protective protein under stress conditions [10]. Overexpression of HSPB1 can enhance the viability of mesenchymal stem cells, showcasing a good therapeutic effect on phosgene-induced acute lung injury [11]. At the same time, HSP70 activates the PI3K/AKT pathway to promote Mesenchymal Stromal Cells survival and migration [12], which also corresponds to the AKT1-HSPB1 edge we found. DNAJ1 and DNAJA4, as HSP70 co-chaperone genes connected to HSPA1B in the key local network, have also been reported in various lung-related diseases, including pulmonary obstruction and hydrogen sulfide exposure[13-15].

HSPAA1 and HSP90AB1, which are closely related to HSPB1, belong to the HSP90 family and play key roles in signal transduction, protein folding, protein degradation, and morphological evolution [16]. As anti-inflammatory partners, HSP90 family proteins may contribute to preventing long-term chronic injuries such as pulmonary fibrosis. Meanwhile, HSP 90 inhibitors may have functional antagonistic effects on short-term and long-term chlorine toxicity [17].

Considering the oxidative nature of phosgene-induced acute lung injury, it is essential to explore antioxidant mechanisms. A high concentration of phosgene can penetrate the pulmonary surfactant layer of alveoli and deplete glutathione, and elevate reactive oxygen species production, causing damage to deeper cells [18]. NQO1, as a phase II antioxidant, plays a certain role in antioxidant activity in acute lung injury [19]. GCLC is crucial in maintaining the glutathione redox status, protecting cells from oxidative stress, and the expression of GCLC increases in the early phase of acute lung injury induced by phosgene [20]. Experiments have shown that various external stimuli, including inhaled particulate matter, significantly increased the gene expression of antagonist enzymes such as GSR and GCLC in mouse alveolar cells [21-24].

The comprehensive analysis of this data underscores the effectiveness of NIEE in predicting imminent lung damage due to phosgene exposure within the first hour. Edges identified in the key local network exhibit robust associations with lung injury and repair, emphasizing the pivotal role of NIEE in early warning and screening for latency following exposure to phosgene.

Note S4

Further analysis of genes in the key local network of LUAD

At the same time, several genes in the key local network of LUAD are worthy of our attention. SFTPA1, SFTPA2, SFTPB, and SFTPC are surfactant genes that can help maintain normal lung function. When these genes are disrupted or mutated, they can cause interstitial pneumonia, pulmonary fibrosis, and other lung diseases and increase the risk of lung cancer by 7 to 14 times [25, 26]. Even worse, they can be associated with atypical bronchiole epithelial hyperplasia [27], which may promote cancer progression and metastasis [28]. In particular, SFTPB and SFTPC were identified as early advanced prognostic markers in LUAD [29], and the suppression of SFTPC expression has been associated with enhanced cell proliferation. It has been identified as a potential predictor of a less favorable survival rate in cases of LUAD [30].

In the key local network (Figure 4E), NAPSA, which is connected to SFTPB and SFTPC, plays a crucial role in proteolytically cleaving pro-SFTPB and pro-SFTPC [31]. Besides, NAPSA is a sensitive and common prognostic marker for LUAD. It is also expressed in a subset of renal cell carcinomas, particularly of the papillary type, as well as in rare cases of papillary thyroid carcinomas [32, 33]. NAPSA may be an effective therapeutic target due to decreased expression in the radiotherapy-resistant group of LUAD patients [34].

In the sub network composed of NEIL3-EXO1 and TOP2A-NEIL3, there are also numerous literature reports on the regulation of genes in the malignant progression of lung cancer, potential prognostic biomarkers, and immune infiltration [35-39]. We believe that the relationships and expression changes of these genes are highly likely to be an important factor in the further irreversible deterioration of LUAD, which is also consistent with the two early-warning signals that NIEE identified.

As for the edge S100P-AGER with the highest score in the key local network, S100P is a member of the S100 family of proteins containing 2 EF-hand calcium-binding motifs. S100 proteins are localized in the cytoplasm and nucleus of a wide range of cells and are involved in the regulation of several cellular processes, such as cell cycle progression and differentiation [40]. LUAD is characterized by an upregulation of S100P, which mediates its function intracellularly but also extracellularly [41] and becomes a driver gene for tumor cells to recruit and polarize tumor-associated macrophages [42]. Besides, S100P plays an important role in the expression profile for molecular diagnosis of NSCLC at the early stage [43], and the overexpression of S100P promotes breast cancer metastasis [44]. Most importantly, S100P is considered a novel therapeutic target for cancer, especially in LUAD metastasis [45]. AGER is a member of the immunoglobulin superfamily of cell surface receptors. It is a multiligand receptor, and besides AGE, interacts with other molecules implicated in homeostasis, development, and inflammation, and certain diseases [40]. It could increase the thickness and permeability of the endovascular membrane in certain pathological conditions [46], and there is some evidence that AGER might be the risk factor and potential biomarker for diagnosis and prognosis targets for LUAD [46-48]. Both S100P and AGER are overexpressed in LUAD and may contribute to tumor progression and metastasis by modulating the tumor microenvironment and immune cell infiltration [49].

From the perspective of cheminformatics, Cromolyn can block the coimmunoprecipitation of S100P with RAGE (AGER) and attenuates the growth of pancreatic tumors and sensitizes tumor cells to gemcitabine (a chemotherapeutic agent) [50, 51]. This evidence indicates that our NIEE

algorithm has potential effectiveness in identifying edges and cancer targets.

Meanwhile, ClusPro (protein-protein docking software) was used to simulate and analyze the protein interaction level of the important edge AGER (PDB: 4LP5) - S100P (PDB: 1J55) [52, 53]. In the largest members cluster with 114 members, the weighted score of center and lowest energy of the two proteins are -795.1 and -1028.4, respectively. These Weighted Scores within the context of ClusPro's typical score ranges could be indicative of a favorable interaction between these two important proteins.

We believe that with the help of NIEE algorithm, existing software and literature, the S100P-AGER edge could be a signature that serves as a potential biomarker in the key local network for predicting early-warning signals, response to potential deterioration or metastasis of LUAD patients and providing stronger evidence in the future therapeutic target.

Note S5

Further exploration - Application of NIEE in destructive insulitis and hyperglycemia of NOD mice with incomplete information

GSE15150 included gene expression of pancreatic lymph nodes of up to 7 individual NOD mice at 6 different time points, with a control group of 20 NOD.B10 mice at 10 days and 20 weeks. In the original research, the onset of destructive insulitis was considered to occur at 12 weeks, leading to the destruction of β cells and overt hyperglycemia in NOD mice [54].

Since the log ratio normalized the dataset, we constructed a normal distribution matrix $(\mu = 1, \sigma = 0.01, n = number\_of\_genes \times 20)$ to represent the expression of NOD.B10 processed signal. Correspondingly, the expression of the NOD processed signal was calculated based on $\mu = 1$.

A background network for Mus musculus was constructed based on StringDB with a threshold of 0.85 to calculate the NIEE score of each experimental mouse. Then, the average NIEE scores at each time point were calculated. Based on the frequency of the top 100 edges with the highest NIEE scores appearing in each sample, we identified edges that appeared at least 17 times (top 50%) in 35 NOD samples, resulting in a key local network (Figure S5C) composed of 18 key edges (Table S11).

Examining the trend of average NIEE scores over time revealed a sharp increase between 10 days and four weeks, reaching its peak at four weeks (Figure S5A). This surge may signify the early-warning signal for destructive islet inflammation emerging as early as four weeks or even earlier. The landscape plot of z-standardized NIEE scores in the fourth week indicated the overall reflection of the key local network on individual biological changes (Figure S5B). Enrichment analysis of genes in this network highlighted their close connection with the endoplasmic reticulum, Golgi apparatus, and pancreatic secretion, with 13 genes confirmed to be related to the pancreas (Figure S5D).

Building on the insights gained from the NIEE analysis of disease induction and progression in NOD mice, we further delved into the key local network identified in our study. Comprising potential genes associated with diabetes development, this network might provide new insights for informing future research.

Among the identified genes, H2-T3 emerged as the gene with the highest degree in the key local network. Fasolino et al. alterations in the chromatin structure and transcriptional expression near the H2-T3 gene in NOD mice, potentially contributing to the induction of diabetes [55].

Another notable gene, PRSS3, a member of the trypsin family of serine protease genes, has been found to be abundantly secreted in obese Zucker rats [56]. In the related research of human homologous genes, PRSS3, known for promoting the growth and metastasis of human pancreatic cancer [57], is also inextricably linked with diabetes [58, 59].

TMED6, identified with implications in diabetes, exhibited a markedly reduced expression in diabetic rats [60]. Knockdown experiments in Min6 β cells demonstrated a consequential reduction in insulin secretion [61]. Additionally, pancreatic islets from individuals with type 2 diabetes exhibited reduced TMED6 expression compared to healthy controls, emphasizing its potential role in maintaining normal pancreatic function and insulin secretion [61]. These findings suggest a potential functional role for TMED6 in islet biology, specifically in hormone production or secretion, and imply that its dysregulation may be associated with diabetes development. Moreover, it is worth

noting that other TMED family genes connected to TMED6 in the subnet of the key local network (Figure S5C) also play an important role in the development of diabetes mellitus [62-64].

Note S6

Further exploration - The impact of NIEE on disease early-warning signals in integrated background networks: A case study of GSE30550.

In order to further investigate the impact of NIEE in other background networks and correlation coefficients, we used String PPI [65], KEGG pathway [66], WikiPathways [67] and Reactome [68] as the integrated background network and switched to the maximum information coefficient [69] to further analyze and evaluate the broad applicability of NIEE in the GSE30550 dataset, respectively.

In details of the experiment to demonstrate the effectiveness of NIEE in the integrated background network, we combined StringDB with KEGG pathways (Influenza A (hsa05164) and other related pathways (e.g., MAKP signaling pathway, Cell cycle, etc.) demonstrated on Influenza A pathway map), WikiPathways (Influenza Infection, TLR3 cascade, OAS antiviral response, Deubiquitination, etc.) and Reactome (R-HSA-168255 Influenza Infection) as the integrated background network.

For consistency and comparison purposes, all the parameter settings were the same as in the previous experiments in Section 3.2 of the main text. We calculated local NIEE difference scores for each subject at 15 time points (Figure S6A). Ultimately, the top 1% edges in the local NIEE difference scores of all symptomatic subjects at each time point were filtered out, and the key local network of high-frequency edges (gene pairs) appearing at least five times at the two highest scoring moments were filtered out based on their frequency of occurrence, totaling 14 edges (Table S12). It is worth noting that in this key local network, 12 edges overlap with the previous key local network in Section 3.2 (Table S1). Next, we considered the sum of the scores of the key local networks as the key differential local NIEE score ($\Delta LH(s+1)$) for further analysis.

In Figure S6A, there was a significant difference in the key differential local NIEE score between subjects with clinical symptoms (red line charts) and asymptomatic subjects (green line charts). A sharp increase in the score indicates the tipping point at which significant changes are about to occur, and for symptomatic subjects, this means that clinical symptoms are about to appear. At the same time, we drew a line chart of NIEE scores for each subject at different times (Figure S6A), as well as a specific NIEE score chart for each symptomatic subject (Figure S6B) and a biological temporary table (Figure S6C) to better illustrate the early-warning signals and onset moment of each symptomatic subject.

Obviously, all symptomatic subjects showed early-warning signals and eight of them showed early-warning signals before the onset of influenza clinical symptoms, while the remaining one symptomatic subject showed early-warning signals near the moment of clinical symptoms onset.

We also compared the early-warning signals obtained from the new integrated network and only String PPI, respectively. We found that only one false positive signal was identified in subject five, detecting from either the new integrated network (Figure S6C) or the original String PPI (Figure 2C). There are similar results to be explored for detecting the early-warning signals from the new integrated network and the original String PPI by comparison between Figure S6 and Figure 2. It means that our method may be independent of the background network to detect the early-warning signals.

Note S7

Further exploration - The impact of NIEE on disease early-warning signals in different correlation coefficients: A case study of GSE30550.

Whereas in the experiment of using other correlation coefficients to measure the correlation between genes, we used the maximum information coefficient, which possesses lower computational complexity and has higher robustness, for the correlation calculation between genes. Specifically, we replaced the Pearson correlation coefficients for calculating the correlation between individual genes in Eqn. (4)-(5) with the maximum mutual information coefficients in the following Eqn. (S1)-(S3)

$$p_{x,y}(s) = \frac{\left|MIC_{x,y}(s)\right|}{\sum_{i=1}^{M}\left|MIC_{x,y}(s)\right|} \quad (S1)$$

$$MIC_{x,y}(s) = \max_{a*b<B} \frac{\int p(x,y)\log_2 \frac{p(x,y)}{p(x)p(y)}dxdy}{\log_2 \min(a,b)} \quad (S2)$$

$$B = n^{0.6} \quad (S3)$$

where the probability coefficients originally composed of PCC will be replaced by MIC. In Eqn. (S2)-(S3), x, y represent the expression of two different genes, $a * b$ represents the number of clumps and n represents the number of samples.

In the case where the background network and parameters are consistent with previous experiments, we ultimately screened out the key local networks of high-frequency edges (gene pairs) with at least nine occurrences at the two highest-scoring moments, totaling 13 edges, based on their frequency of occurrence (Table S13). We consider the sum of scores for the key local network as the key differential local NIEE score ($\Delta LH(s+1)$) for further analysis.

Similar to the previous section, in Figure S7A, there was a significant difference in the key differential local NIEE score between subjects with clinical symptoms (red line charts) and asymptomatic subjects (green line charts). A sharp increase in the score indicates the tipping point at which significant changes are about to occur, and for symptomatic subjects, this means that clinical symptoms are about to appear. And the line chart of NIEE scores for each subject (Figure S7A), specific NIEE score chart for each symptomatic subject (Figure S7B) and a biological temporary table (Figure S7C) were plotted to illustrate the early-warning signals better.

Among the results of MIC, eight subjects' early-warning signals appeared before the onset of influenza clinical symptoms, while the early-warning signal of symptomatic subject 5 appeared near the onset of clinical symptoms. In the results, all symptomatic subjects showed early-warning signals and the early-warning signals calculated using the MIC as a correlation coefficient (Figure S7) were similar to the results from PCC (Figure 2).

It means that NIEE is robust, and different correlation methods do not easily impact the time points of the early-warning signals.

Note S8

Further exploration - The impact of the genes' weights in NIEE on disease early-warning signals: A case study of GSE30550.

In the analysis of the early stages of carcinogenesis, Remacle F. et al. introduced molecular weight as an important coefficient in calculating entropy [70]. Building upon this insight, we have further discussed the weights of edge information entropy and variance in the NIEE algorithm. Here, we used the protein sequence length corresponding to all genes in the Uniprot database [71] as weights for NIEE score calculation.

Specifically, different from Eqn. (1)-(2) where the degrees of each node (gene) are considered as weights, we proposed the protein sequence length corresponding to the gene as the weight of that node. Furthermore, the information entropy and variance values of the edges should be calculated from the arithmetic mean of the weights of the two corresponding nodes in Eqn. (S4)-(S5),

$$H_E(s) = \frac{\alpha}{\alpha+\beta} \cdot H_{g_1}(s) + \frac{\beta}{\alpha+\beta} \cdot H_{g_2}(s) \qquad (S4)$$

$$SD_E(s) = \frac{\alpha}{\alpha+\beta} \cdot SD_{g_1}(s) + \frac{\beta}{\alpha+\beta} \cdot SD_{g_2}(s) \qquad (S5)$$

where α, β represent the protein sequence length of the corresponding node (gene) respectively.

In the case where the background network and other parameters are consistent with previous experiments, we screened out the key local networks of high-frequency edges (gene pairs) with at least four occurrences at the highest scoring moments, totaling 13 edges, based on their frequency of occurrence (Table S15). We consider the sum of scores for the key local network as the key differential local NIEE score ($\Delta LH(s+1)$) for further analysis.

A significant difference in the key differential local NIEE score was observed between subjects with clinical symptoms (red line charts) and asymptomatic subjects (green line charts), as shown in Figure S8A. A sharp increase in the score indicated the tipping point at which significant changes were imminent. For symptomatic subjects, this meant that the appearance of clinical symptoms was about to occur. Concurrently, a line chart was generated to depict the NIEE scores for each subject at various points in time (Figure S8A). Furthermore, a chart specific to each symptomatic subject (Figure S8B) and a biological temporal table (Figure S8C) were created to illustrate the early-warning signals and onset moment of each symptomatic subject more effectively. It is evident that all symptomatic subjects exhibited early-warning signals, with 7 of them exhibiting such signals prior to the onset of influenza-related clinical symptoms. In contrast, the remaining two symptomatic subjects exhibited early-warning signals in the immediate vicinity of the clinical symptom onset.

The key local network was subjected to enrichment analysis, the results of which are presented in Table S16. The analysis revealed that the majority of genes are associated with immune-related functions such as Cytosolic DNA-sensing pathway, NOD-like receptor signaling pathway, Influenza, and virus. It is worth noting that the sub-network in the key local network, including CCL8, CCR1, CCR5 and CXCL10, highly overlaps with the key local network we previously proposed in Table S1. This may suggest that this sub-network plays an important role in influenza early warning. Additionally, another hub gene, GBP1, despite not being included in the existing influenza A pathway, has been demonstrated to play a vital role in anti-influenza viral

activity as the interferon-inducible protein [72]. Moreover, GBP1 transcript and protein were significantly upregulated when response to the infection by influenza A virus, and GBP1 was found to interact directly with the viral nonstructural protein 1 (NS1) [73], which is an accessory function protein during viral infection [74], denoting that it may be a pivotal gene in the early stage of influenza A infection.

The preceding results and analysis indicate that the protein sequence length may not be as effective as the degree in serving as an early-warning signal for disease. However, it can still provide insights into disease warning and key gene networks.

Note S9

Further comparison - The effect of BioTIP and NIEE on disease early-warning signals: A case study of TCGA-LUAD.

The BioTIP algorithm represents a noteworthy advancement in the realm of disease early warning systems. It has enhanced the correlation-based tipping-point models by incorporating a shrinkage estimation approach to large-scale correlation matrices, thereby facilitating the detection of non-chaotic critical transition signals (CTSs).

Due to inaccessible to the vignette tutorial of BioTIP (https://bioconductor.org/packages/release/bioc/vignettes/BioTIP/inst/doc/BioTIP.html), we had to operate commands from the November 2023 archive in the Internet Archive Wayback Machine (https://web.archive.org).

In the specific data processing process, we selected the TCGA-LUAD dataset consistent with Section 3.3 of the main text and grouped samples according to disease stage (Table S8). At the same time, we marked the paracancerous tissue samples (58 in total) in Stage IA as a separate "Para" group to enable comparative analysis.

During the gene selection phase, specifically within the “sd_selection(df, samplesL, cutoff)” procedure, we experimented with cutoff values of 0.01 and 0.05 respectively. The biological modules identified at each disease stage are depicted in Figures S9A and S10A. After module filtration, the progression tendencies of each state, as indicated by clusters with the highest Module Contribution Index (MCI) scores, are illustrated in Figures S9B and S10B. And the MCI simulation scores, which are indicative of the magnitude of CTS, are exhibited in Figures S9C and Figure S10C. The density distribution of random IC scores, generated by shuffling sample labeling, is displayed in Figures S9E and S10E.

In the experiment with cutoff values of 0.01, the highest DNB scores group is “Stage IV” (Figure S9B), and the highest IC scores group is “Stage IV” (Figure S9D). For the experiment with cutoff values of 0.05, the highest DNB scores group is “Para” (Figure S10B), and the high IC scores group are “Para” and “Stage IV” (Figure S10D).

Upon analyzing the results, it is evident that the BioTIP algorithm, while innovative, did not perform optimally for early disease warning in the TCGA-LUAD dataset. In contrast, our NIEE algorithm demonstrated superior performance, providing clear early-warning signals indicative of cancer progression (in Stage IB) and metastasis (in Stage IIIB) with powerful literature support. This distinction underscores the robustness and potential of the NIEE algorithm as a more effective tool for early detection and intervention in disease progression.

Reference

1. Zaas AK, Chen M, Varkey J et al. Gene expression signatures diagnose influenza and other symptomatic respiratory viral infections in humans, Cell Host Microbe 2009;6:207-217.
2. Jackson GG, Dowling HF, Spiesman IG et al. Transmission of the common cold to volunteers under controlled conditions. I. The common cold as a clinical entity, AMA Arch Intern Med 1958;101:267-278.
3. Turner RB. Ineffectiveness of intranasal zinc gluconate for prevention of experimental rhinovirus colds, Clin Infect Dis 2001;33:1865-1870.
4. Wang X, Hinson ER, Cresswell P. The Interferon-Inducible Protein Viperin Inhibits Influenza Virus Release by Perturbing Lipid Rafts, Cell Host & Microbe 2007;2:96-105.
5. Chin KC, Cresswell P. Viperin (cig5), an IFN-inducible antiviral protein directly induced by human cytomegalovirus, Proc Natl Acad Sci U S A 2001;98:15125-15130.
6. Sciuto AM, Phillips CS, Orzolek LD et al. Genomic analysis of murine pulmonary tissue following carbonyl chloride inhalation, Chem Res Toxicol 2005;18:1654-1660.
7. Duniho SM, Martin J, Forster JS et al. Acute changes in lung histopathology and bronchoalveolar lavage parameters in mice exposed to the choking agent gas phosgene, Toxicol Pathol 2002;30:339-349.
8. Chen P, Liu R, Li Y et al. Detecting critical state before phase transition of complex biological systems by hidden Markov model, Bioinformatics 2016;32:2143-2150.
9. Liu R, Chen P, Chen L. Single-sample landscape entropy reveals the imminent phase transition during disease progression, Bioinformatics 2020;36:1522-1532.
10. Parseghian MH, Hobson ST, Richieri RA. Targeted heat shock protein 72 for pulmonary cytoprotection, Ann N Y Acad Sci 2016;1374:78-85.
11. Jin C, Zhou F, Zhang L et al. Overexpression of heat shock protein 70 enhanced mesenchymal stem cell treatment efficacy in phosgene-induced acute lung injury, J Biochem Mol Toxicol 2020;34:e22515.
12. Kim JE, Ko AR, Hyun HW et al. P2RX7-MAPK1/2-SP1 axis inhibits MTOR independent HSPB1-mediated astroglial autophagy, Cell Death Dis 2018;9:546.
13. Qiu XB, Shao YM, Miao S et al. The diversity of the DnaJ/Hsp40 family, the crucial partners for Hsp70 chaperones, Cell Mol Life Sci 2006;63:2560-2570.
14. Nitika, Blackman JS, Knighton LE et al. Chemogenomic screening identifies the Hsp70 co-chaperone DNAJA1 as a hub for anticancer drug resistance, Sci Rep 2020;10:13831.
15. Faust O, Abayev-Avraham M, Wentink AS et al. HSP40 proteins use class-specific regulation to drive HSP70 functional diversity, Nature 2020;587:489-494.
16. Chen B, Piel WH, Gui L et al. The HSP90 family of genes in the human genome: insights into their divergence and evolution, Genomics 2005;86:627-637.
17. Neumeister SM, Gray JP. The Strategic National Stockpile: identification, support, and acquisition of medical countermeasures for CBRN incidents, Toxicol Mech Methods 2021;31:308-321.
18. Lu Q, Huang S, Meng X et al. Mechanism of Phosgene-Induced Acute Lung Injury and Treatment Strategy, Int J Mol Sci 2021;22.
19. Reddy AJ, Christie JD, Aplenc R et al. Association of human NAD(P)H:quinone oxidoreductase 1 (NQO1) polymorphism with development of acute lung injury, J Cell Mol Med 2009;13:1784-1791.

20. Ji L, Liu R, Zhang XD et al. N-acetylcysteine attenuates phosgene-induced acute lung injury via up-regulation of Nrf2 expression, Inhal Toxicol 2010;22:535-542.
21. Li N, Nel AE. Role of the Nrf2-mediated signaling pathway as a negative regulator of inflammation: implications for the impact of particulate pollutants on asthma, Antioxid Redox Signal 2006;8:88-98.
22. Rouse RL, Murphy G, Boudreaux MJ et al. Soot nanoparticles promote biotransformation, oxidative stress, and inflammation in murine lungs, Am J Respir Cell Mol Biol 2008;39:198-207.
23. Li YJ, Takizawa H, Azuma A et al. Disruption of Nrf2 enhances susceptibility to airway inflammatory responses induced by low-dose diesel exhaust particles in mice, Clin Immunol 2008;128:366-373.
24. Zhang H, Liu H, Davies KJ et al. Nrf2-regulated phase II enzymes are induced by chronic ambient nanoparticle exposure in young mice with age-related impairments, Free Radic Biol Med 2012;52:2038-2046.
25. Honda T, Sakashita H, Masai K et al. Deleterious Pulmonary Surfactant System Gene Mutations in Lung Adenocarcinomas Associated With Usual Interstitial Pneumonia, JCO Precis Oncol 2018;2:1-24.
26. Shang J, Song Q, Yang Z et al. Identification of lung adenocarcinoma specific dysregulated genes with diagnostic and prognostic value across 27 TCGA cancer types, Oncotarget 2017;8:87292-87306.
27. Wang Y, Kuan PJ, Xing C et al. Genetic defects in surfactant protein A2 are associated with pulmonary fibrosis and lung cancer, Am J Hum Genet 2009;84:52-59.
28. Vachani A, Nebozhyn M, Singhal S et al. A 10-gene classifier for distinguishing head and neck squamous cell carcinoma and lung squamous cell carcinoma, Clin Cancer Res 2007;13:2905-2915.
29. Wang L, Liu W, Liu K et al. The dynamic dysregulated network identifies stage-specific markers during lung adenocarcinoma malignant progression and metastasis, Molecular Therapy - Nucleic Acids 2022;30:633-647.
30. Li B, Meng Y-Q, Li Z et al. MiR-629-3p-induced downregulation of SFTPC promotes cell proliferation and predicts poor survival in lung adenocarcinoma, Artificial Cells, Nanomedicine, and Biotechnology 2019;47:3286-3296.
31. Johansson J, Jörnvall H, Curstedt T. Human surfactant polypeptide SP-B Disulfide bridges, C-terminal end, and peptide analysis of the airway form, FEBS Letters 1992;301:165-167.
32. Bishop JA, Sharma R, Illei PB. Napsin A and thyroid transcription factor-1 expression in carcinomas of the lung, breast, pancreas, colon, kidney, thyroid, and malignant mesothelioma, Hum Pathol 2010;41:20-25.
33. He D, Wang D, Lu P et al. Single-cell RNA sequencing reveals heterogeneous tumor and immune cell populations in early-stage lung adenocarcinomas harboring EGFR mutations, Oncogene 2021;40:355-368.
34. Gao J, Lu F, Yan J et al. The role of radiotherapy-related autophagy genes in the prognosis and immune infiltration in lung adenocarcinoma, Front Immunol 2022;13:992626.
35. Kou F, Sun H, Wu L et al. TOP2A Promotes Lung Adenocarcinoma Cells' Malignant Progression and Predicts Poor Prognosis in Lung Adenocarcinoma, J Cancer 2020;11:2496-2508.
36. Zhou CS, Feng MT, Chen X et al. Exonuclease 1 (EXO1) is a Potential Prognostic Biomarker and Correlates with Immune Infiltrates in Lung Adenocarcinoma, Onco Targets Ther 2021;14:1033-1048.

37. Guo W, Sun S, Guo L et al. Elevated TOP2A and UBE2C expressions correlate with poor prognosis in patients with surgically resected lung adenocarcinoma: a study based on immunohistochemical analysis and bioinformatics, J Cancer Res Clin Oncol 2020;146:821-841.
38. Zhao C, Liu J, Zhou H et al. NEIL3 may act as a potential prognostic biomarker for lung adenocarcinoma, Cancer Cell Int 2021;21:228.
39. Huang H, Hua Q. NEIL3 Mediates Lung Cancer Progression and Modulates PI3K/AKT/mTOR Signaling: A Potential Therapeutic Target, Int J Genomics 2022;2022:8348499.
40. Maglott D, Ostell J, Pruitt KD et al. Entrez Gene: gene-centered information at NCBI, Nucleic Acids Res 2007;35:D26-31.
41. Rehbein G, Simm A, Hofmann H-S et al. Molecular regulation of S100P in human lung adenocarcinomas, International Journal of Molecular Medicine 2008.
42. Wu J, Zhou J, Xu Q et al. Identification of Key Genes Driving Tumor Associated Macrophage Migration and Polarization Based on Immune Fingerprints of Lung Adenocarcinoma, Frontiers in Cell and Developmental Biology 2021;9.
43. Bartling B, Rehbein G, Schmitt WD et al. S100A2–S100P expression profile and diagnosis of non-small cell lung carcinoma: Impairment by advanced tumour stages and neoadjuvant chemotherapy, European Journal of Cancer 2007;43:1935-1943.
44. Du M, Wang G, Barsukov IL et al. Direct interaction of metastasis-inducing S100P protein with tubulin causes enhanced cell migration without changes in cell adhesion, Biochem J 2020;477:1159-1178.
45. Arumugam T, Logsdon CD. S100P: a novel therapeutic target for cancer, Amino Acids 2011;41:893-899.
46. Liu W, Ouyang S, Zhou Z et al. Identification of genes associated with cancer progression and prognosis in lung adenocarcinoma: Analyses based on microarray from Oncomine and The Cancer Genome Atlas databases, Molecular Genetics & Genomic Medicine 2018;7.
47. Stav D, Bar I, Sandbank J. Usefulness of CDK5RAP3, CCNB2, and RAGE genes for the diagnosis of lung adenocarcinoma, Int J Biol Markers 2007;22:108-113.
48. Zhang W, Fan J, Chen Q et al. SPP1 and AGER as potential prognostic biomarkers for lung adenocarcinoma, Oncol Lett 2018;15:7028-7036.
49. Yang J, Chen X, Lin M et al. Advanced Glycation End Products Receptor DNA Methylation Is Associated with Immune Infiltration and Prognosis of Lung adenocarcinoma and lung squamous cell carcinoma, Research Square 2021.
50. Arumugam T, Ramachandran V, Sun D et al. Designing and developing S100P inhibitor 5-methyl cromolyn for pancreatic cancer therapy, Mol Cancer Ther 2013;12:654-662.
51. Kim CE, Lim SK, Kim JS. In vivo antitumor effect of cromolyn in PEGylated liposomes for pancreatic cancer, J Control Release 2012;157:190-195.
52. Ormo M, Cubitt AB, Kallio K et al. Crystal structure of the Aequorea victoria green fluorescent protein, Science 1996;273:1392-1395.
53. Kozakov D, Hall DR, Xia B et al. The ClusPro web server for protein-protein docking, Nat Protoc 2017;12:255-278.
54. Kodama K, Butte AJ, Creusot RJ et al. Tissue- and age-specific changes in gene expression during disease induction and progression in NOD mice, Clin Immunol 2008;129:195-201.
55. Fasolino M, Goldman N, Wang W et al. Genetic Variation in Type 1 Diabetes Reconfigures the 3D Chromatin Organization of T Cells and Alters Gene Expression, Immunity 2020;52:257-

274.e211.
56. Habara Y, Uehara A, Takasugi Y et al. Characterization of secretory responses in exocrine pancreas of genetically obese Zucker rats, International journal of pancreatology 1991;10.
57. Jiang G, Cao F, Ren G et al. PRSS3 promotes tumour growth and metastasis of human pancreatic cancer, Gut 2010;59:1535-1544.
58. Bandesh K, Bharadwaj D. Genetic variants entail type 2 diabetes as an innate immune disorder, Biochimica et Biophysica Acta (BBA) - Proteins and Proteomics 2020;1868.
59. Kuo C-S, Chen J-S, Lin L-Y et al. Inhibition of Serine Protease Activity Protects Against High Fat Diet-Induced Inflammation and Insulin Resistance, Scientific Reports 2020;10.
60. Wang X, Yang R, Jadhao SB et al. Transmembrane Emp24 Protein Transport Domain 6 is Selectively Expressed in Pancreatic Islets and Implicated in Insulin Secretion and Diabetes, Pancreas 2012;41:10-14.
61. Fadista J, Vikman P, Laakso EO et al. Global genomic and transcriptomic analysis of human pancreatic islets reveals novel genes influencing glucose metabolism, Proceedings of the National Academy of Sciences 2014;111:13924-13929.
62. Zhou L, Li H, Yao H et al. TMED family genes and their roles in human diseases, International Journal of Medical Sciences 2023;20:1732-1743.
63. Kim S-H, Lee E-S, Yoo J et al. Predicting risk of type 2 diabetes mellitus in Korean adults aged 40–69 by integrating clinical and genetic factors, Primary Care Diabetes 2019;13:3-10.
64. Li T, Yang F, Heng Y et al. TMED10 mediates the trafficking of insulin-like growth factor 2 along the secretory pathway for myoblast differentiation, Proceedings of the National Academy of Sciences 2023;120.
65. Szklarczyk D, Gable AL, Nastou KC et al. The STRING database in 2021: customizable protein-protein networks, and functional characterization of user-uploaded gene/measurement sets, Nucleic Acids Res 2021;49:D605-D612.
66. Kanehisa M, Furumichi M, Sato Y et al. KEGG for taxonomy-based analysis of pathways and genomes, Nucleic Acids Res 2023;51:D587-D592.
67. Pico AR, Kelder T, van Iersel MP et al. WikiPathways: pathway editing for the people, PLoS Biol 2008;6:e184.
68. Milacic M, Beavers D, Conley P et al. The Reactome Pathway Knowledgebase 2024, Nucleic Acids Res 2024;52:D672-D678.
69. Reshef DN, Reshef YA, Finucane HK et al. Detecting novel associations in large data sets, Science 2011;334:1518-1524.
70. Remacle F, Kravchenko-Balasha N, Levitzki A et al. Information-theoretic analysis of phenotype changes in early stages of carcinogenesis, Proc Natl Acad Sci U S A 2010;107:10324-10329.
71. UniProt C. UniProt: the Universal Protein Knowledgebase in 2023, Nucleic Acids Res 2023;51:D523-D531.
72. Nordmann A, Wixler L, Boergeling Y et al. A new splice variant of the human guanylate-binding protein 3 mediates anti-influenza activity through inhibition of viral transcription and replication, FASEB J 2012;26:1290-1300.
73. Zhu Z, Shi Z, Yan W et al. Nonstructural protein 1 of influenza A virus interacts with human guanylate-binding protein 1 to antagonize antiviral activity, PLoS One 2013;8:e55920.
74. Hale BG, Randall RE, Ortin J et al. The multifunctional NS1 protein of influenza A viruses, J

Gen Virol 2008;89:2359-2376.
